# Composites, Fabrication and Application of Polyvinylidene Fluoride for Flexible Electromechanical Devices: A Review

**DOI:** 10.3390/mi11121076

**Published:** 2020-12-03

**Authors:** Shuaibing Guo, Xuexin Duan, Mengying Xie, Kean Chin Aw, Qiannan Xue

**Affiliations:** 1 State Key Laboratory of Precision Measuring Technology & Instruments, College of Precision Instrument and Opto-Electronics Engineering, Tianjin University, Tianjin 300072, China; 2019202091@tju.edu.cn (S.G.); xduan@tju.edu.cn (X.D.); mengying_xie@tju.edu.cn (M.X.); 2 Department Mechanical Engineering, University of Auckland, Auckland 1023, New Zealand; k.aw@auckland.ac.nz

**Keywords:** PVDF, piezoelectric polymer, wearable device, flexible sensor, electromechanical

## Abstract

The technological development of piezoelectric materials is crucial for developing wearable and flexible electromechanical devices. There are many inorganic materials with piezoelectric effects, such as piezoelectric ceramics, aluminum nitride and zinc oxide. They all have very high piezoelectric coefficients and large piezoelectric response ranges. The characteristics of high hardness and low tenacity make inorganic piezoelectric materials unsuitable for flexible devices that require frequent bending. Polyvinylidene fluoride (PVDF) and its derivatives are the most popular materials used in flexible electromechanical devices in recent years and have high flexibility, high sensitivity, high ductility and a certain piezoelectric coefficient. Owing to increasing the piezoelectric coefficient of PVDF, researchers are committed to optimizing PVDF materials and enhancing their polarity by a series of means to further improve their mechanical–electrical conversion efficiency. This paper reviews the latest PVDF-related optimization-based materials, related processing and polarization methods and the applications of these materials in, e.g., wearable functional devices, chemical sensors, biosensors and flexible actuator devices for flexible micro-electromechanical devices. We also discuss the challenges of wearable devices based on flexible piezoelectric polymer, considering where further practical applications could be.

## 1. Introduction

Piezoelectric materials have been in development for 140 years [1]. These materials are widely used in electromechanical devices because they can convert mechanical energy to electrical energy under pressure or generate mechanical motion by electricity [2,3]. The use of piezoelectric materials for the preparation of micro-electromechanical devices makes it possible to develop miniature resonators [4,5,6,7,8], frequency meters [9] and micro energy supply chips [10,11,12,13,14]. Piezoelectric crystals have a single crystal structure and natural piezoelectricity, such as α-quartz [15,16]. In addition to natural piezoelectric materials that can adapt to different application scenarios, researchers have also manufactured many high-performance artificial piezoelectric materials. Typical piezoelectric-crystal materials include aluminum nitride (AlN) and zinc oxide (ZnO) [17,18], which have a variety of advantages, such as very high strength, large piezoelectric coefficients and good applicability to micro devices [19,20]. Piezoelectric ceramics consist of many small crystals and the crystals are randomly oriented. The crystal orientation can be changed through a polarization process to exhibit piezoelectricity. The polarization process is usually performed by applying a high electric field. PZT-series materials such as PMN-PZT and PMMPT are typical representatives of piezoelectric ceramics; they not only have high piezoelectric coefficients but also good ductility. However, the bending property of PZT-series materials is still poor, and they pollute the environment because of their lead content. Generally, these inorganic piezoelectric materials have the features of high hardness and brittleness, which are not suitable for flexible devices that require high material flexibility. 

With the rapid development of wearable devices, there is an increasing demand for device flexibility. Compared with inorganic and ceramic materials, piezoelectric polymers have more remarkable bending and tensile properties. Polymer piezoelectric materials have a carbon chain as the backbone, and their flexibility is higher than those of single crystals and ceramics [21,22,23]. This high flexibility allows them to withstand a greater amount of strain, thus making them more suitable for application scenarios with large bending and twisting requirements. Some suitable piezoelectric polymers, such as polyvinylidene fluoride (PVDF), polyvinylidene fluoride-trifluoro ethylene (P(VDF-TrFE)), cellulose and its derivatives, polyamides and polylactic acids, are widely used in pressure sensors, vibrometers, audio sensors, ultrasonic sensors, impact sensors, display equipment, piezoelectric catalysis for wastewater treatment [24,25], chemical sensors and medical sensors [26]. Among flexible piezoelectric materials, PVDF has a high piezoelectric coefficient, good flexibility and excellent biocompatibility; therefore, PVDF is widely used to develop flexible electromechanical devices.

However, improvement of the polarization characteristics of flexible piezoelectric materials continues to be challenging. The piezoelectric coefficient of PVDF can be effectively improved by doping with inorganic piezoelectric materials, such as PZT and piezoelectric ceramics. In addition, nano-clay, BTO, Ag, graphene and other nanomaterials can change the crystal structure of PVDF and increase the β phase proportion. Moreover, some new fabrication methods can also improve the polarity of flexible piezoelectric materials. In this paper, the recent progress in material science of PVDF and PVDF composites, aiming for micro fabrication techniques, is reviewed. We discuss the latest technological scheme and processing methods and compare their merits and demerits. The improvement in the performance of flexible piezoelectric materials has overcome the limitations of wearable electromechanical devices. The applications of these materials in electromechanical devices are discussed in detail. Finally, the development trend of flexible electromechanical devices based on PVDF is prospected in the future, such as using the help of piezoelectric properties of biocrystals, improving the biocompatibility for the development of bionic devices.

## 2. Polyvinylidene Fluoride-Based-Series Piezoelectric Material

### 2.1. The Piezoelectric Effect Principle of PVDF

The piezoelectric effect [27,28] refers to the polarization of certain dielectrics in a specific direction under the action of an external force. A dielectric material has positive and negative charges on two opposite surfaces. When the external force is eliminated, the material returns to a neutral state. This phenomenon is called the positive piezoelectric effect. As the direction of the force changes, the polarity of the charge also changes. Conversely, when an electric field is applied in the polarization direction of the dielectric, the material will deform. When the electric field is removed, the deformation disappears. This phenomenon is called the reverse piezoelectric effect. The fundamental reason for the piezoelectric effect is that the mechanical deformation caused by the polarization intensity is linear with respect to the electric field strength in crystal materials without a symmetry center.

Piezoelectric materials have unique mechanical and electrical coupling characteristics, including the direct piezoelectric effect of charge generated under the action of external mechanical stress and the converse piezoelectric effect of mechanical strain caused by an external electric field. They are usually expressed by the following piezoelectric coupling equation [29]:

(1) $$\left[\begin{array}{c} \delta \\ D \end{array}\right]=\left[\begin{array}{cc} s^{E} & d\\ d & \varepsilon^{\sigma } \end{array}\right]\left[\begin{array}{c} \sigma \\ E \end{array}\right]$$

For the direct piezoelectric effect, electric displacement *D* caused by stress σ based on piezoelectric effect and the internal electric field E based on material’s dielectric permittivity. Here, $D=d\sigma +\varepsilon^{\sigma }E$, where *d* is the piezoelectric coefficient and $\varepsilon^{\sigma }$ is the dielectric permittivity for constant stress. For the converse piezoelectric effect, strain δ is caused by stress σ and the internal electric field based on converse piezoelectric effect. Here, $\delta =s^{E}\sigma +dE$, where $s^{E}$ is the elastic compliance for constant electric field. The direct piezoelectric effect is very important for the sensing and energy collection of surface charge generated by applied stress on piezoelectric materials. The electromechanical actuator of electro-deformation is based on the inverse piezoelectric effect.

Many piezoelectric materials have a very clear polar axis. Energy harvesting performance is related to the angle between the stress and polar axis. The direction of the polar axis is Direction 3, and the direction perpendicular to the polar axis is Direction 1. The direction of stress application can be along either Direction 1 or 3 [30].

**Figure 1:**
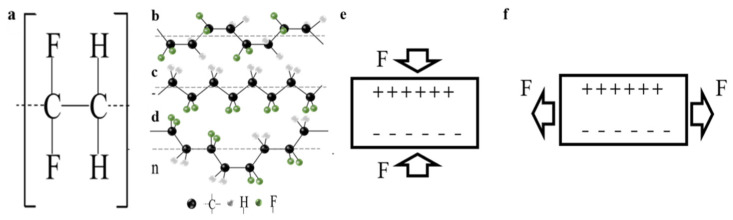
(**a**) Structure of PVDF; (**b**) space structure of α-phase PVDF; (**c**) space structure of β-phase PVDF; (**d**) space structure of γ-phase PVDF; and the two transition mechanisms of PVDF (**e**) Mode 33 and (**f**) Mode 31.

The piezoelectric properties of PVDF are directly related to its structure [31]. PVDF is a semi-crystalline polymer polymerized by H_2_C =CF_2_ monomer, whose properties depend on the phase due to the alignment of crystals (Figure 1a). When two CF_2_ or CH_2_ groups are connected, a defect occurs in the connected polymer chain. Defects in the polymeric chain influence the polarity of the PVDF film, thereby increasing the piezoelectric response. The polymer has α, β, γ, б and ε phases, which are five semi-crystalline polymorphs. The α and β phases are the most common phases. The γ phase, which is not common, is a transitional state between α and β. б and ε are difficult to isolate. The α phase of PVDF is non-polar [32], because it contains two molecular chains and the dipole moments are reversed, forming a trans-gauche conformation (TGTG′) (Figure 1b). The β phase of PVDF has an all-trans conformation (TTTT) (Figure 1c). Most hydrogen atoms and fluorine atoms are separated; therefore, it has a dipole moment perpendicular to the polymer chain, which shows the highest net dipole moment. The γ phase of PVDF is a transitional state between α and β (Figure 1d), and it has a smaller dipole moment than the β phase [33]. It is necessary to increase the proportion of β phase in PVDF to maximize its electromechanical conversion efficiency [34]. PVDF has two transition mechanisms: Mode 31 is perpendicular to the direction of stress applied to the material, and Mode 33 is parallel to the direction of stress [26]. These two transition mechanisms are shown in Figure 1e,f.

### 2.2. Piezoelectric Materials based on PVDF and Its Derivatives

Polymer piezoelectric material is a material with a carbon chain as the basic skeleton, and its flexibility is higher than that of single crystals and ceramics [21,22]. This high flexibility allows it to withstand a greater amount of strain, thereby making it more suitable for application scenarios with large bending and twisting requirements. Piezoelectric polymer PVDF and its copolymers have the advantages of natural flexibility, easy processing and sufficient mechanical strength, which make them more suitable for flexible sensors than inorganic piezoelectric materials [35,36,37,38,39,40,41]. 

**Figure 2:**
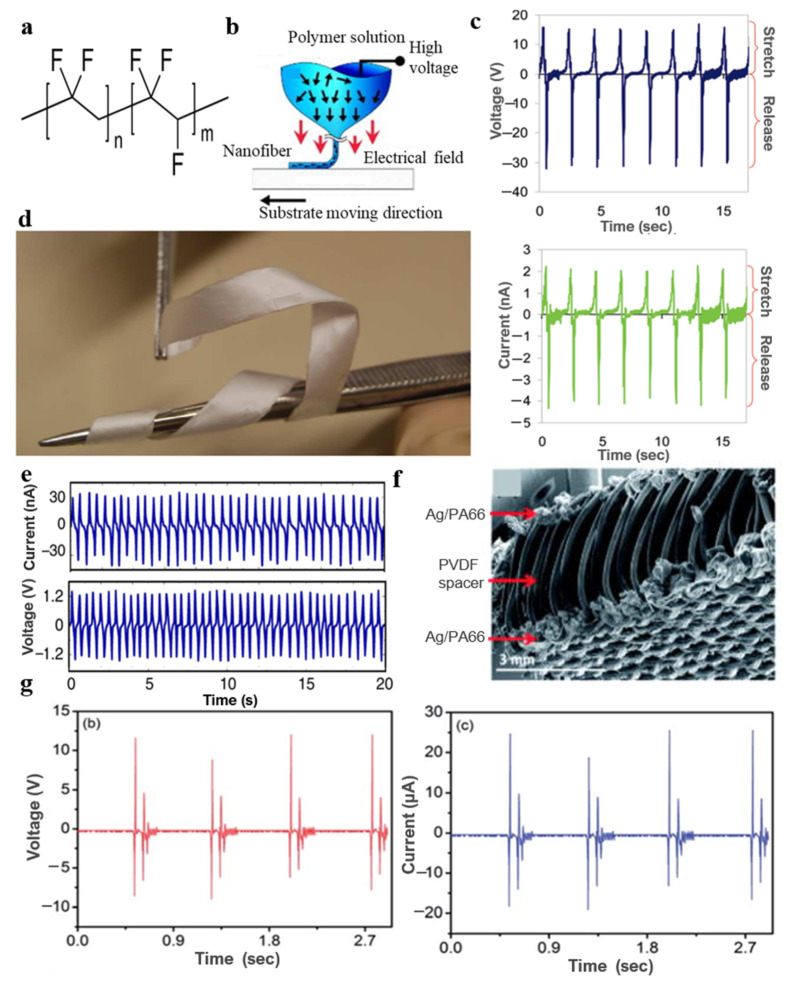
(**a**) Structure of P(VDF-TrFE); (**b**) PVDF nanofibers with the high component of β-phase PVDF by near-field electrospinning [41]; (**c**) the generator based on fiber could provide a maximum output power is 5–30 mV and 0.5–3 nA with different feature sizes [41]; (**d**) a free-standing film’s photograph and SEM image [42]; (**e**) the output power of the generators based on textile [42]; (**f**) the “3D spacer” cross-sectional SEM image [50]; and (**g**) 2D and 3D piezoelectric textile’s output power [50].

Thus far, PVDF has been found to exist as five semi-crystalline polymorphs: α, β, γ, б and ε. Among these, α phase is not electroactive, and β phase has the strongest piezoelectricity. PVDF with a low component of β phase has a d33 value of approximately 20–30 pC/N [42,43,44,45,46,47,48,49]. Therefore, increasing the β-phase PVDF composition is vital to enlarge the mechanical–electrical energy conversion performance of PVDF. Therefore, the piezoelectric coefficient of PVDF is lower than that of common inorganic piezoelectric materials. Chang et al. [41] prepared PVDF nanofibers with a high β-phase PVDF component by near-field electrospinning (Figure 2a). The fiber-based generator could provide a maximum output voltage of 30 mV and maximum output current of 3 nA (Figure 2c). It has a much higher energy conversion efficiency than generators made of PVDF thin films [41]. 

The P(VDF-TrFE) copolymer has high crystallinity and piezoelectric properties, thus it has a high piezoelectric response [51,52,53]. Persano et al. [42] proposed a piezoelectric textile based on highly aligned electrospun P(VDF-TrFE) fibers. The proposed piezoelectric textile has a large area, and it is flexible and freestanding (Figure 2d) [42]. The maximum open-circuit voltage of the generators based on textiles can reach 1.5 V (Figure 2e) [42], thus showing excellent flexibility and mechanical strength. Many new designs have emerged in the practice field to produce all-fiber piezoelectric textiles. A “3D spacer” based on all-fiber piezoelectric textiles was reported [50], consisting of top and bottom electrodes and a spacer yarn. The spacer yarn is composed of high concentration of β-phase PVDF monofilaments. The top and bottom electrodes are composed of silver-coated polyamide multifilament yarn layers (Figure 2f) [50]. The new type of 3D textile-based PVDF generator had a much larger output power density than the traditional 2D piezoelectric textile, up to 5.07 μW/cm^2^ (Figure 2g) [50].

### 2.3. Piezoelectric Materials Doped in PVDF

**Figure 3:**
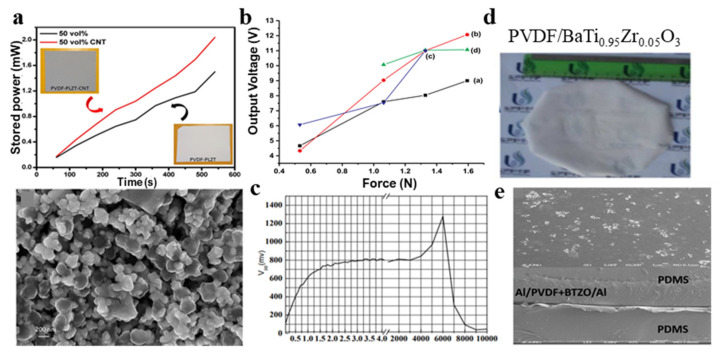
(**a**) Variation of stored power with time and SEM of PLZT particles dispersed in the PVDF matrix [56]; (**b**) the relationship between output voltage and applied forces with different frequencies [57]; (**c**) the relationship between output frequency and voltage of PVDF nanofiber composite containing BNT-ST [9]; (**d**) optical diagram PVDF/BTZO hybrid film [61]; and (**e**) SEM of PVDF/BTZO hybrid film [61].

To balance the contradiction between flexibility and higher voltage coefficient, researchers have tried to mix polymer piezoelectric materials with inorganic piezoelectric materials. Because of the good piezoelectric properties and relatively soft properties of PZT and the tensile and flexural properties of PVDF, the combination of the two will make sense. According to the above ideas, Tiwari et al. [54] prepared PZT and PVDF piezoelectric composite films using solution casting technology. This method can achieve both electric field polarization and mechanical stretching during the electrospinning process, making it very suitable for manufacturing piezoelectric nanofibers. Similarly, the dielectric properties of PZT/P (VDF-TrFE) composite films [55] were also studied. In 2020, Pal et al. [56] used lanthanum-doped lead zirconate titanate (PLZT), PVDF and multi-walled carbon nanotubes (MWCNTs) as supplementary fillers to create a three-phase hybrid piezoelectric nanogenerator. Figure 3a shows the variation of stored power with time and SEM of PLZT particles dispersed in the PVDF matrix. Raad et al. [57] fabricated nanogenerator devices on the basis of composite structure of PVDF, ZnO, nanorods and BaTiO_3_. The voltage output of these composite structures is up to 12 V under the force of 1.5 N (Figure 3b).

Considering the compatibility with the human body, lead-free materials are more popular. Titanate or bismuth [58] are mixed with PVDF to develop lead-free piezoelectric nanofiber composites. The piezoelectric properties of PVDF can be changed by adding calcined BaTiO_3_ ceramic powder [59,60]. 

Researchers at Kyungnam University synthesized an excellent lead-free piezoelectric material [9]. It is a kind of flexible lead-free piezoelectric nanofiber composite of BNT-ST((1−*x*)Bi0.5Na0.5TiO_3−_ *x*SrTiO) ceramics and PVDF polymer, which was fabricated by electrospinning. By measuring the functional relationship between the output frequency and voltage of the PVDF nanofiber composite containing BNT-ST (Figure 3c) [9], the piezoelectric properties of the composite and the sensitivity of the piezoelectric output to frequency are substantially improved because BNT-series materials can overcome large coercive field defects and exhibit strong piezoelectric properties [62,63,64,65,66].

In 2015, Alluri et al. [61] developed a flexible hybrid film of PVDF doped with highly crystalline BaTi(1−*x*)ZrxO_3_ (*x* = 0, 0.05, 0.1, 0.15 and 0.2) nanocomposites (BTZO). During preparation, the PVDF matrix solution was embedded in BTZO using a molten-salt process under ultrasonication (Figure 3d) [61]. The SEM images of the PVDF/BTZO hybrid film are shown in Figure 3e. It can be concluded that, by the molten salt method, replacing the Ti4+ (0.605 Å) site with a Zr4+ atom (0.72 Å) can modify the high-purity bulk BaTiO_3_ nanocomposites and enhance the piezoelectric coefficient (d33) from 100 pC/N (BaTiO_3_) [67,68,69,70,71] to 174–236 pC/N (BaTi(1−*x*)ZrxO_3_) [72,73,74,75,76]. The properties of BTZO/PVDF hybrid films have been substantially improved compared to those of pure piezoelectric films and have great potential in manufacturing environmental protection devices, active sensors and flexible nanogenerators.

### 2.4. Conductive Nanomaterials Doped in PVDF

**Figure 4:**
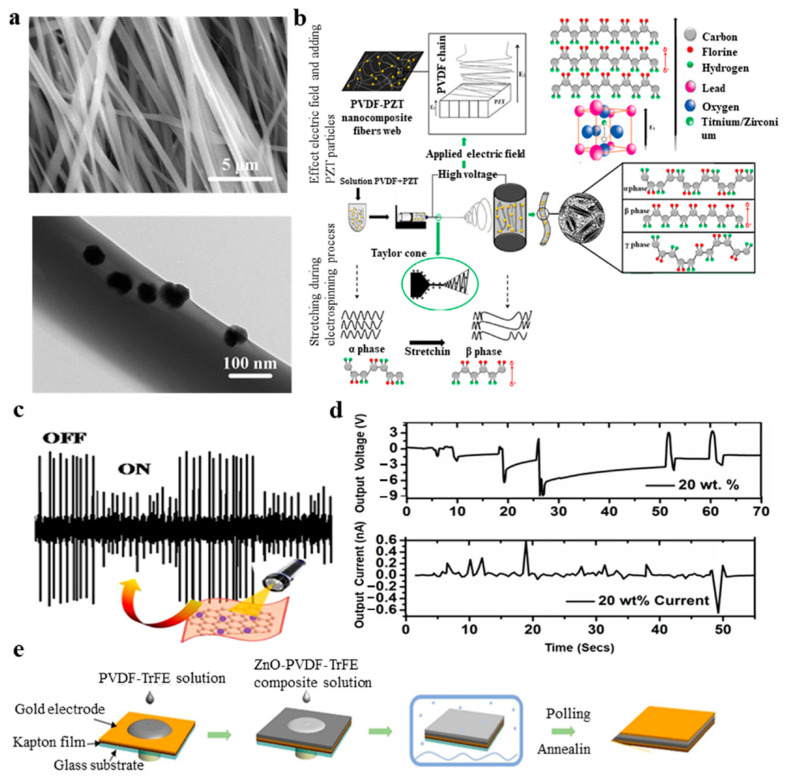
(**a**) The SEM of BTO/P(VDF-TrFE) nanocomposite fiber [90]; (**b**) PVDF-PZT nanocomposite fiber, the crystallization mechanism and β-phase formation based on adding PZT [11]; (**c**) change of piezoelectric coefficient in the form of output voltage under different light conditions [98]; (**d**) output voltage and current signal of GFO-PVDF composite films [108]; and (**e**) schematic of the manufacturing process of the device [112].

Although polymer materials have the advantages of high flexibility and high mechanical strength compared to inorganic materials, the piezoelectric coefficient of piezoelectric materials is relatively small; moreover, their piezoelectric conversion efficiency is low, and they do not easily conduct electricity. The introduction of nanomaterials may change the crystal structure of polymer piezoelectric polymers and improve the piezoelectric properties [77,78,79,80,81,82]. Zirconate titanate, barium titanate and zinc oxide have high piezoelectric constants; therefore, polymer piezoelectric matrices usually use them as piezoelectric fillers [83,84,85,86,87,88]. Compared with those of the pure piezoelectric polymer, the piezoelectric properties of the nanocomposites are improved, dielectric constant is significantly increased, crystallinity of the phase is increased and local stress is increased. Adding nanoclay [89] to PVDF can prompt the formation of the β-phase in the spinning process, which is an ideal method to prepare piezoelectric nanofibers. BTO nanoparticles were used to improve the P(VDF-TrFE) nanofibers’ piezoelectric sensing properties (Figure 4a) [90]. BTO/P(VDF-TrFE) nanofibers doped with BTO nanoparticles have the largest β-phase crystallinity and the best piezoelectric properties.

Wu et al. [91] studied the piezoelectric properties, crystal structure and sound absorption properties of PVDF nanofibers doped with carbon nanotubes prepared by electrospinning. The results show that adding CNTs further improves the piezoelectric properties and the ability to absorb sound waves at low frequencies. Hosseini et al. [92] studied the potential synergistic effect of Cloisite 30B (OMMT) nanoclay and multi-walled carbon nanotube (MWCNT) nanofillers on PVDF crystal structure and piezoelectric device performance. They evaluated PVDF fiber mats’ sound-absorbing and piezoelectric properties, and the results show that, compared with OMMT, the MWCNT could decrease PVDF impedance and increase the dielectric constant. MWCNT/OMMT hybrid nanocomposites have good sound absorption properties, which might be caused by the enhanced interaction at the polymer-filled interface between the acoustic wave and OMMT plates and MWCNT nanotubes [93,94,95,96,97]. They found that the sound absorption efficiency of PVDF/MWCNT/OMMT hybrid nanocomposites was higher than that of pure PVDF fibers and film. Negar et al. [11] fabricated PVDF nanocomposites by doping with PZT particles. The PVDF-PZT nanocomposite fiber, the crystallization mechanism and β-phase formation based on the addition of PZT are shown in Figure 4b.

PVDF and graphene-silver (GAg) nanocomposites have plasma-coupled piezoelectric properties, and the piezoelectric energy conversion efficiency reaches 15% [98]. Using the unique interface structure of silver nanoparticles, silver-doped oriented PVDF nanofibers with a high content of β phase were prepared by electrospinning [99], and the crystallinity of the β phase was 44.5%. Silver nanoparticles in graphene dispersion form n-type doping on graphene owing to electrostatic effects [100,101,102,103,104]. The self-polarization of silver nanoparticles in PVDF is beneficial to the nucleation of the electroactive β phase. PVDF nanocomposites are environmentally friendly and low-cost nanocomposites, having high piezoelectric coefficients and different piezoelectric coefficients under different light conditions (Figure 4c) [98].

Gan et al. [105] developed composite materials using TiO_2_ nanoparticles and PVDF. The increase in conductivity makes the polarization process easier, and the required electric field was reduced from 260 to 120 MV/m. In 2017, Al-Saygh et al. [106] proposed a flexible pressure sensor based on PVDF doped with rGO and TiO_2_ nanolayers (TNL). The hybrid PVF/rGO-TNL film had a higher β-phase content (75.68%) than PVDF (70.37%), PVDF/rGO (72.73%) and PVDF/TNL (73.5%) films. Similarly, Pusty et al. [107] combined iron with carbon nanotubes and graphene nanomaterials and proposed that adding CNT and Fe-RGO to PVDF can increase the conductivity of nanocomposite membranes. Mishra et al. [108] developed a flexible piezoelectric polymer nanocomposite film to collect mechanical energy, which is based on PVDF doped with gallium ferrite nanoparticles (GFO). Under mechanical pressure and release conditions, the output voltage and current are 3.5 V and 4 nA, respectively (Figure 4d). As a type of piezoelectric ceramic, nano ZnO is also used as a piezoelectric filler [109]. Dodds et al. [110] prepared PVDF TrFE/ZnO nanoparticle films by spin coating and studied their piezoelectric response. The prepared films not only maintain their mechanical flexibility but also improve their piezoelectric properties. Zinc oxide-PVDF composite membrane [111] can also be prepared by sol-gel technology. The latest report is on the preparation of metal ZnO PVDF composite films by doping ZnO nanoparticles into metals, which shows the potential for mechanical energy generation and motion sensing. Figure 4e shows the fabrication process of the device. They studied and compared the voltage output of pure ZnO-PVDF samples and ZnO-PVDF composites doped with sodium (Na), cobalt (Co), silver (Ag), ferric oxide (Fe_3_O_4_) and lithium (Li) [112].

### 2.5. Biomaterials Functional PVDF

**Figure 5:**
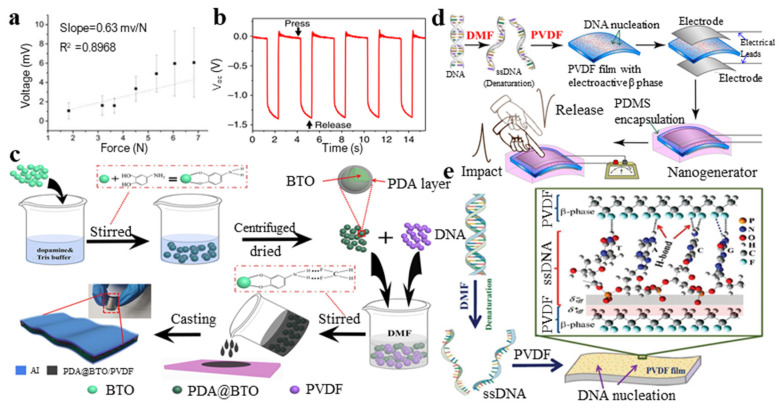
(**a**) Piezoelectric voltage generated increases with increasing applied force [114]; (**b**) open-circuit voltage of the FF-generator [115]; (**c**) the fabrication process of PDA@BTO/PVDF composite membrane on the basis of flexible pressure sensor [116]; (**d**) DNA was used as a nucleating agent to produce a self-polarized PVDF membrane to fabricate nanogenerator [117]; and (**e**) denaturation of DNA and the forming process of ß-enriched DNA-PVDF membrane [117].

Some biomaterials have certain piezoelectric properties naturally. In 2017, Nuraeva et al. [113] investigated (S)-glutamine and ortho-carboranyl derivatives of (S)-asparagine films and bulk crystal piezoelectric properties, demonstrating a very high piezoelectric response. The high local transverse piezoelectric coefficients of these biocrystals indicate that they are promising materials for various piezoelectric applications. Stapleton et al. [114] provided experimental evidence of the globulin and lysozyme’s direct piezoelectric effect. They measured the direct piezoelectric effect of the lysozyme crystal aggregation film and found that the average piezoelectric coefficients of the monoclinic and tetragonal lysozyme films were 0.94 and 3.16 pC/N, respectively (Figure 5a). Nguyen et al. [115] reported that diphenylalanine (FF) is a short peptide composed of two natural amino acids, with special piezoelectric properties and excellent mechanical properties (Figure 5b). Doping with a piezoelectric polymer can stimulate the piezoelectric properties. Recently, Yang et al. [116] modified barium titanate (BTO) with polydopamine (PDA). Then, the mixing ratio was adjusted between it and the PVDF matrix, and they finally formed a uniform and homogeneous PDA@BTO/PVDF composite material. The facial solution-casting method was used to make the flexible piezoelectric pressure sensor shown in Figure 5c. PDA can improve the dispersion of BTO in the PVDF matrix and reduce interface void defects and cracks between the two components. The output voltage is about 2 times higher than the unmodified one and 13.3 times higher than the original PVDF. Tamang et al. [117] reported that DNA was used as a nucleating agent to produce a self-polarized PVDF membrane with higher piezoelectric properties (Figure 5d). This nucleating agent can achieve the molecular dipole arrangement and the nucleation of the electroactive β phase in PVDF. Phosphate ions interact with hydrogen bonds on the single-strand deoxyribonucleic acid (ss-DNA) backbone to form a stable polar β phase, accounting for more than 80% of polar β phase in the PVDF matrix (Figure 5e).

## 3. Fabrication and Polarization of PVDF

There are many methods to convert PVDF into the β phase, including electrospinning, spin coating, solvent casting and 3D printing. PVDF processed by spin coating and solvent casting methods mainly forms the α phase; therefore, a subsequent polarization method is needed to increase the β phase state of PVDF [118,119]. Polarization can be achieved with a set of widely used techniques that can be used to reorient polymers to increase the net polarization vector in three directions, which is an important step during the fabrication process. For the processing methods of electrospinning and 3D printing, the polarization process can be completed directly in situ under the action of an electric field and temperature, and no subsequent polarization process is required. The following sections mainly discuss the preparation and polarization methods of PVDF films, including the piezoelectric effect principle.

### 3.1. Fabrication of PVDF by Spin Coating and Solvent Casting

**Figure 6:**
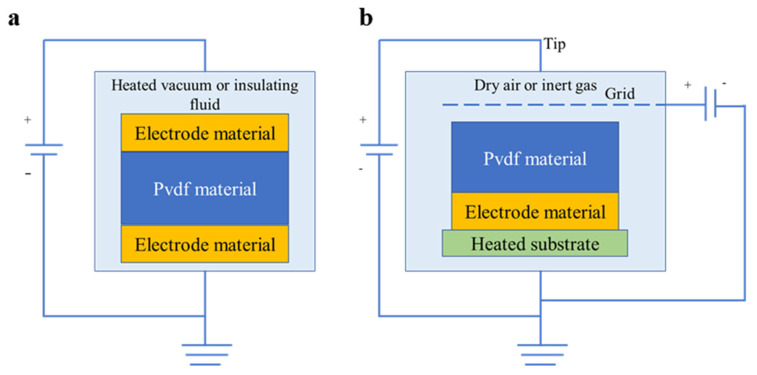
(**a**) Schematic of electrode poling method; and (**b**) corona poling method.

Spin coating and solvent casting mainly form the α phase of PVDF; therefore, a subsequent polarization process is required. In the spin coating method, the surface stress is increased during the spinning process. The addition of some compounds helps induce and contribute to the formation of the β phase state of PVDF, and sometimes it can be formed with a certain piezoelectric coefficient without subsequent polarization. A schematic diagram of the spin coating and solvent casting is shown in Figure 6a,b.

**Figure 7:**
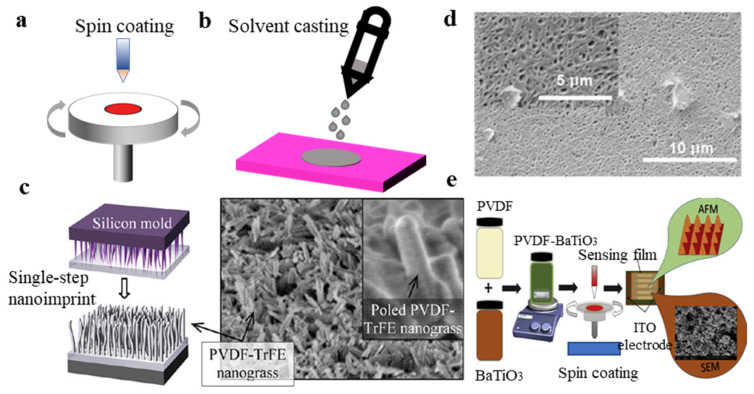
(**a**) Schematic diagram of the spin coating; (**b**) schematic diagram of the solvent casting; (**c**) spin-coated PVDF-TrFE was dissolved in MEK and the silicon mold was hot pressed on the film [120]; (**d**) SEM of PVDF–TrFE copolymer thin films fabricated by spin coating [121]; and (**e**) preparing the PVDF-BaTiO_3_ nanocomposite films by spin coating [123].

Electrode poling and corona poling are the two most common methods of polarization. Electrode poling has the advantages of high d33 coefficient and reproducibility. Electrode poling is the relatively simpler one of these two methods. The PVDF film is sandwiched between two electrodes and then wrapped by a shell (Figure 6a). In the process of corona poling, there is a heated substrate at the bottom of the shell and an electrode on the heated substrate. The PVDF film is only in contact with one of the electrodes. The corona tip is placed on the top and applied with a 10 kV voltage. There is a grid between tip and PVDF material. The grid is applied a voltage, which is much lower than 10 kV. Therefore, there is a voltage difference close to 10 kV between corona tip and grid. It ionizes the gas between the corona tip and the grid. The ionized gas accelerates to the film, and the PVDF film is polarized (Figure 6b). Corona poling needs to keep the gas in the shell dry. The electron beam poling method uses a focused electron beam and irradiates the PVDF film to reorient into the β phase. In addition to the above polarization methods, mechanical drawing and additive manufacturing can polarize PVDF. As for additive manufacturing, we can induce the formation of the β phase using composite systems. Table 1 shows the advantages of various polarization methods. The method of solvent casting mainly relies on the addition of some compounds and application of high temperature and external electric field, to promote the formation of the β phase state of PVDF. Chien et al. [120] spin-coated PVDF–TrFE dissolved in MEK and hot embossed the silicon mold on the film under DC poling (Figure 7c). The electric voltage varied from 30 to 60 V, and the temperature was 90 or 110 °C. The maximum piezoelectricity of the single PVDF−TrFE nanopillar was 210.4 pm/V, with an average d33 value of 72.7 pC/N of the developed PVDF−TrFE nanograss structures. Tushar et al. [121] spin-coated PVDF–TrFE copolymer into thin films to induce the formation of the β phase with a d33 value varying from 38 to 74 pC/N (Figure 7d). Schulze et al. [122] prepared P(VDF-TrFE) films for 20 µm thickness by solvent casting under the effect of electrode poling and an electric field of 75 MV/m. The d33 coefficients were measured to be about 20 pC/N. The spin-coating technique [123] was used to prepare the PVDF-BaTiO_3_ nanocomposite films on an interdigital ITO electrode(Figure 7e). Vineet et al. [54] fabricated PVDF/PZT piezoelectric composite films by a solution cast technique. The d33 value varied from 60 to 84 pC/ N. The higher is the content of PZT ceramic, the greater is the proportion of β phase of the PVDF.

After the PVDF film is formed, there are further studies to constrain the nanostructure of the film, such as the serpentine structure [124,125,126] and Kirigami structure [127] formed by cutting plotter, achieving sufficient compliance of mechanical deformation to skin motion. Conversely, the polymer film is cut into the desired shape and then stacked and laminated to increase the density of the filler [128].

### 3.2. Fabrication of PVDF by Electrospinning

**Figure 8:**
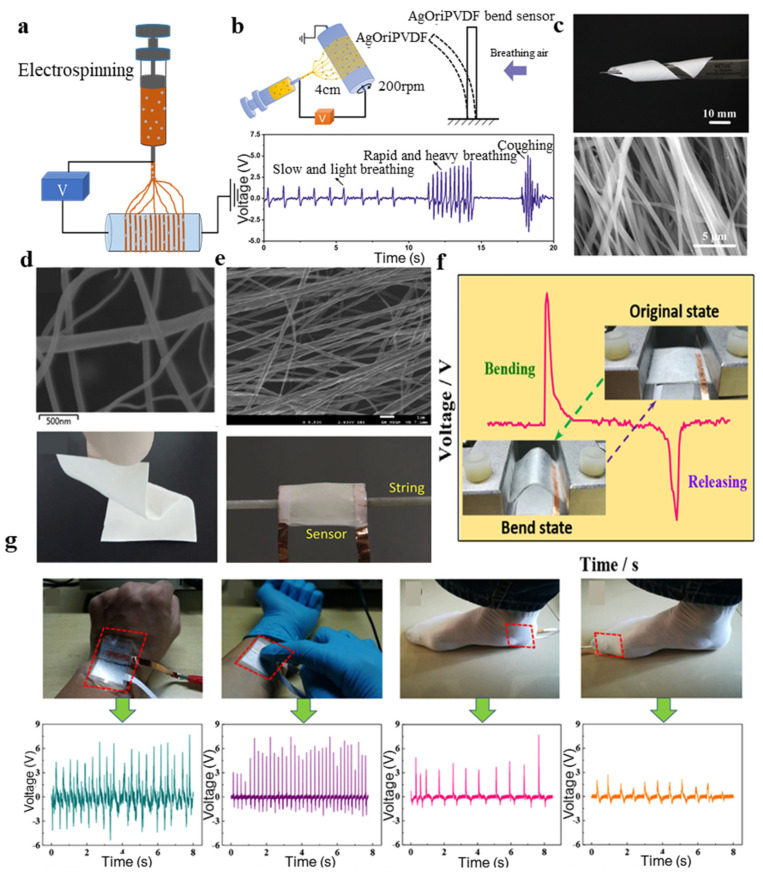
(**a**) Schematic diagram of the Electrospinning technique; (**b**) flexible bend sensor to monitor human respiration [99]; (**c**) optical photo and SEM of (BTO)/P(VDF-TrFE) composite nanofibers [90]; (**d**) optical photo and SEM of BNT-ST/PVDF nanofiber composite [9]; (**e**) PVDF strain sensor for measuring the force of the string [131]; (**f**) mechanical movement in the primitive phase and the bending phase [132]; and (**g**) optical image and output voltage generated by human movement [132].

Electrospinning is a very promising processing technology. The needle tube containing the PVDF solution sprays the PVDF solution onto a drum through a nozzle, the substrate wraps around the drum, and a voltage between 10 and 20 kV is usually added between the nozzle and the substrate [129]. Nano- to micro-scale PVDF fibers are randomly deposited on the substrate, forming a low-density PVDF film. The electrospinning technique completes the PVDF deposition and polarization process in one step. The rotation of the drum increases the stress between PVDF and the substrate, which in turn promotes the formation of the β-phase state of PVDF. A schematic diagram of the electrospinning technique is shown in Figure 8a. Gong et al. [130] combined the PVDF nanofiber membrane by electrospinning with PDMS-Ag NWS and PET/ITO electrodes to fabricate sensors. Lu et al. [99] made a flexible bend sensor to monitor human respiration by electrospinning PVDF and a silver mixed solution (Figure 8b).

Xiaohe et al. [90] fabricated (BTO)/P(VDF-TrFE) composite nanofibers by electrospinning and were able to distinguish and sense the movement of walking ants and the energy of a free-falling ball as low as 0.6 μJ (Figure 8c). Sang et al. [9] fabricated flexible lead-free piezoelectric nanofibers composed of PVDF and BNT-ST ceramic by electrospinning. The output voltage measurement system is a frequency function for the BNT-ST/PVDF nanofiber composite module (Figure 8d). Rahul et al. [131] fabricated PVDF nanofibers with a high β-phase state to develop a PVDF strain sensor to measure the string force (Figure 8e). Kunming et al. [132] fabricated nanocomposite fiber mats by electrospinning graphene nanosheets, barium titanate and PVDF, obtaining as high as 11 V of open-circuit voltage (Figure 8f,g).

### 3.3. Fabrication of PVDF Devices by 3D Print

**Figure 9:**
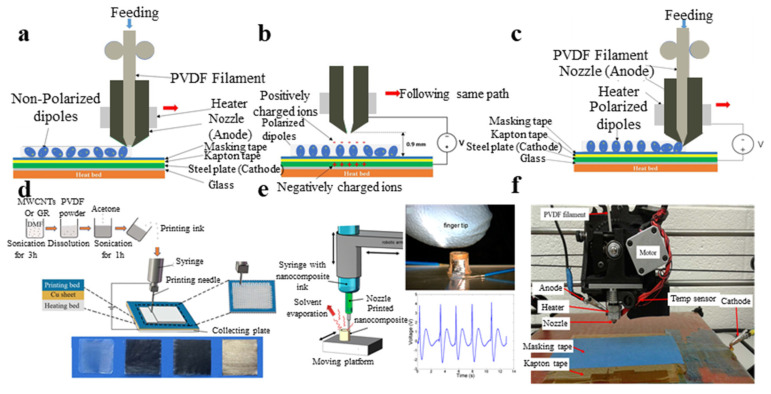
(**a**) Schematic of 3D printing of PVDF layer; (**b**) corona poling process; (**c**) simultaneous 3D printing and in-situ polarization; (**d**) 3D printing process and fabricated PVDF film [133]; (**e**) 3D cylindrical sensor’s piezoelectric voltage output when five finger taps [134]; and (**f**) optical diagram of 3D printing technique and corona poling process [135].

The 3D print can deposit PVDF or PVDF compounds on the bottom heating plate under heating and nozzle extrusion (Figure 9a). Based on the 3D printing principles, we can incorporate polarizing processes including heat press, electric field poling and mechanical stretching simultaneously. The polarization process is shown in Figure 9b.

Finally, we completed the integration of 3D printed PVDF and PVDF composites with the in situ polarized PVDF. The schematic is shown in Figure 9c. The shear force between the nozzle and PVDF, as well as heating, nucleation on filler surfaces and electric field, all help to promote the formation of the β-phase state of PVDF. Hoejin et al. [136] fabricated PVDF/BaTiO_3_ nanocomposites using a 3D printing technique. Then, the composite material was thermally polarized to improve the piezoelectric response of the composite. The piezoelectric response of the PVDF/BaTiO_3_ nanocomposite was three times higher than that of the nanocomposites fabricated by solvent casting. Using the 3D printing technique, we can obtain a more homogeneous dispersion of PVDF/BaTiO_3_ nanocomposites, in comparison with nanocomposites fabricated by solvent casting. Chen et al. [133] fabricated electroactive PVDF thin films of bi-axially orientation using layer-by-layer 3D printing. Carbon nanotubes were added as a nucleating agent to induce the formation of the β-phase state of PVDF (Figure 9d). 

Sampada et al. [134] fabricated flexible, lightweight and complex-shaped piezoelectric devices by 3D printing (Figure 9e). The d31 coefficients were measured to be about 18 pC/N. PVDF films with enhanced β-phase percentage [135] were fabricated by 3D printing (Figure 9f). 

This section reviews the common preparation methods for PVDF. The different fabrication methods, polarization, description and comparison of the performance of PVDF are shown in Table 2. The electrospinning process has the advantages of direct polarization, while spin coating and pouring methods are simpler and more compatible with other processes. As a new technology, 3D printing has also attracted the attention of researchers. Table 2 shows that the electrospinning method still shows higher piezoelectric conversion performance for energy acquisition and strain sensing. However, for the electro deformation, the spin coating method can produce thinner films with greater deformation.

## 4. Flexible Electromechanical Device Made by PVDF

PVDF polymer material has the advantages of low weight, high flexibility, high sensitivity, good fit and ductility and high piezoelectric coefficient. It is suitable for flexible sensors with high flexibility requirements and is used in physics, chemistry and biology. These fields have a wide range of applications.

There are many applications based on PVDF materials, such as energy harvesting device, physical sensors, chemical sensors and biosensors. When PVDF is used for energy harvesting devices and physical sensors, PVDF is mainly used to convert external mechanical energy into electrical energy. Methods such as doping inorganic piezoelectric and nanomaterials and improving processes have been adopted to improve the piezoelectric conversion ability of materials. Piezoelectric polymer-based chemical sensors and biosensors, such as humidity, gas and DNA sensors, are constructed by modifying the sensitive layer on piezoelectric materials, which can specifically adsorb humidity, gas and biomolecules. After specific adsorption of the target, the sensor will undergo a slight volume change, which will cause deformation of the PVDF material, thereby generating electrical signals, or the quality change will cause the sensor’s resonance frequency to shift. The materials, fabrication methods and sensitivity are shown in Table 3.

### 4.1. Energy Harvesting Device

**Figure 10:**
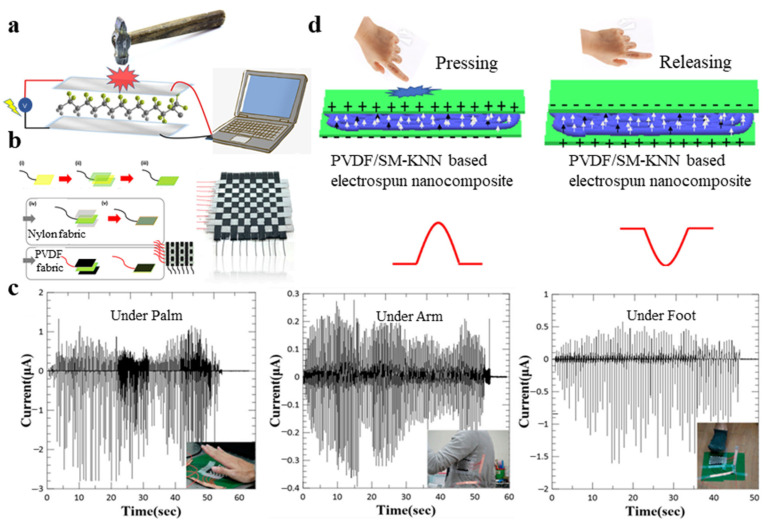
(**a**) Schematic of the energy harvesting device; (**b**) schematic diagram of woven nanogenerator production process [140]; (**c**) nanogenerator harvests energy from human finger tapping, human arm movement and human footsteps [140]; and (**d**) the phenomenon of charge separation during the process of press nanogenerator and release pressure of nanogenerator [141].

The main principle of energy harvesting device manufacturing is to use the piezoelectric conversion effect of piezoelectric materials. Under external pressure, the piezoelectric material deforms and generates a voltage, thereby converting mechanical to electrical energy. A schematic diagram of the energy harvesting device is shown in Figure 10a. 

In 2020, Mokhtari et al. [142] fabricated a high-performance hybrid piezofiber composed of a PVDF and barium titanate (BT) nanoparticle (mass ratio 10:1). These fibers are knitted to fabricate a wearable energy generator with a power density of 87 μW cm^−3^ and a maximum voltage output of 4 V. They confirmed through experiments that the stability performance of the triaxial braided piezoelectric fibers in the bending test during 1000 cycles to a maximum strain of 50% at 0.6 Hz with no change in its performance. In 2019, Sang et al. [143] prepared a wearable piezoelectric energy harvester based on core–shell piezoelectric yarns prepared by twining the yarns around a conductive thread, improving the durability of the energy harvesting process. The yarns were composed of flexible piezoelectric nanofibers of BNT-ST and PVDF-TrFE by electrospinning. Muhammad et al. [140] fabricated a woven nanogenerator using commercial nylon cloth and PVDF nanofibers, which can harvest energy from human motions. A woven nanogenerator harvests energy from human movement; a schematic diagram of the production process of this device is shown in Figure 10b. Figure 10c shows that the nanogenerator harvests energy from human finger tapping, human arm movement and human footsteps. Satyaranjan et al. [141] fabricated a nanogenerator based on flexible PVDF/SM-KNN, which has a current density of 5.5 mA/cm^2^ and a power density of 115.5 mw/cm^2^. They found that the output voltage error of repeated detection under the same pressure is about 1%. Figure 10d shows the charge separation phenomenon during the pressing process and the release pressure of the nanogenerator. 

Researchers at the University of Texas at San Antonio have developed a self-charging pacemaker based on PVDF piezoelectric films [144]. The kinetic energy of the heartbeat is converted into electrical energy to charge the battery. The work is expected to be developed into products on the market within five years.

### 4.2. Physical Sensors

**Figure 11:**
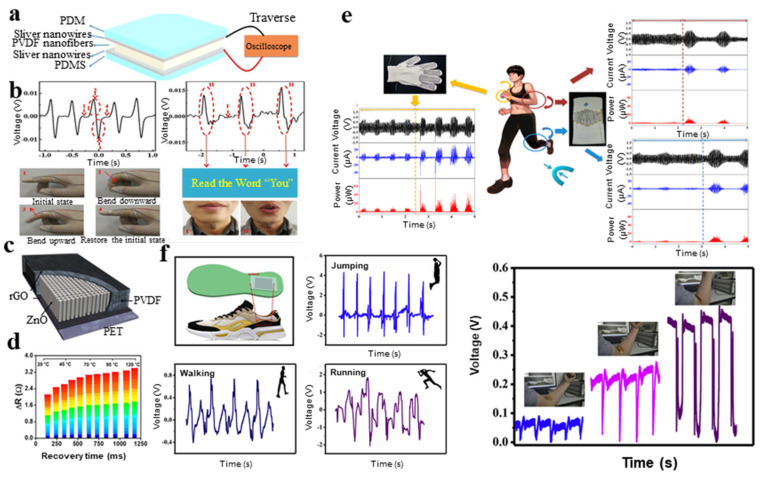
(**a**) Schematic of flexible pressure sensor used to detect pressure signal [130]; (**b**) device was used as a motion sensor to monitor knuckle bends and vocal cord vibrates [130]; (**c**) schematic of sensor based on PVDF and ZnO nanostructures on graphene electrodes [87]; (**d**) the relationship between temperature and recovery time [87]; (**e**) generated output voltages, output currents and output powers by finger bending as well as knee and elbow movements [143]; and (**f**) pressure sensor embedded in the bottom of the insole or stuck on human arm and voltage generated by jumping, walking, running and different bending angles’ elbows [116].

In sensing applications, flexible and wearable pressure sensors are made of piezoelectric polymer materials with flexible characteristics. High-voltage electrospinning can be used to fabricate PVDF piezoelectric nanofiber films [49,145,146,147,148,149]. Gong et al. [130] combined the PVDF nanofiber membrane by electrospinning with PDMS-Ag NWS and PET/ITO electrodes to fabricate sensors. When the tester repeats the word “you”, the detected output signal is also periodic (Figure 11a) [130]. This type of sensor has evident piezoelectric properties and performs well in quantitative pressure measurement. In addition, because of their shape-preserving function, they can be used as real-time monitors of people’s activities (Figure 11b). Lee et al. [87] proposed a highly sensitive gauge sensor based on PVDF and ZnO nanostructures on graphene electrodes (Figure 11c) [87]. It can detect pressure changes with the lowest value of 10 Pa, which is 1000 times lower than the minimum required for artificial skin. The temperature value calculation is based on the signal recovery time (Figure 11d). The PVDF-TrFE copolymer membrane pressure sensor is manufactured via a standard photolithography process that can be used for batch processing, thereby reducing costs. The resulting membrane has good uniformity and high polymer pattern resolution [150]. Alluri et al. [61] processed piezoelectric films made of PVDF and BaTi(1−x)ZrxO_3_, which can effectively convert pressure signals into electrical signals, with a maximum peak power of 15.8 nW. They confirmed superb reproducibility and durability of the PNGs by a stability test using a cyclic pushing instrument. Sang et al. [143] manufactured a wearable piezoelectric energy harvester by electrospinning PVDF-TrFE and BNT-ST(0.78Bi0.5Na0.5TiO_3_-0.22SrTiO_3_). The generated output voltages, output currents and output powers by finger bending as well as knee and elbow movements are shown in Figure 11e. The 3D printed PVDF with BaTiO_3_ (BTO) filler [151] can also be used for pressure sensing. Compared with the single PVDF composite of TNL [152,153,154], the sensitivity was increased by 333.46% at 5 kPa under the effects of the new additives shape, good interaction and well-distributed hybrid additives in the matrix. This confirmed that it was possible to fabricate low-cost and lightweight electronic devices with reduced quantities of metal oxides and pressure sensing devices.

Ye et al. [116] fabricated flexible wearable pressure sensors that were sensitive to various human behaviors based on piezoelectric materials. The pressure sensor was embedded in the bottom of the insole or stuck on the human arm, and the voltage generated by jumping, walking, running and elbows at different bending angles is shown in Figure 11f. According the fatigue testing of the sensor for 1000 cycles at 12 N, the output voltages dropped by 6.7%, demonstrating the excellent stability and durability of the sensor. 

Commercially, many commercial products related to pressure are based on PVDF materials. For example, based on PVDF, Dymedix Diagnostics, Inc (Minnesota, USA) developed airflow and snore sensors [155]. Cambridge Touch Technologies (CCT) is a fast-growing high-tech enterprise originating from the University of Cambridge. Its KF Piezo series PVDF is a well-known piezoelectric film product in the industry. CCT has developed an UltraTouch Sensor with a simple structure, a low cost and a high performance [156].

### 4.3. Chemical Sensor based on Piezoelectric Polymer

As a flexible piezoelectric material, the mechanical deformation state of PVDF electrically driven is directly related to its mass load. Then, by modifying the sensor surface with adsorbent materials, it can specifically adsorb gas or water vapor to form a mass sensor. For example, when the sensing interface contacts the target gas, the physical shape and mass changes caused by the absorption of gas will affect the resonance frequency and other physical parameters of the piezoelectric polymer-sensitive film. Zengwei et al. [157] applied the phase separation method to deposit a PVDF microporous membrane on the surface of a palladium-loaded tin dioxide (Pd-SnO_2_) gas-sensitive film. The stability of the sensor under high humidity was evidently improved by the influence of the PVDF microporous membrane. To detect the hydrogen concentration, a hydrogen sensor [158] can be made by sticking the Pd film on both sides of the PVDF membrane. Because of the selective absorption of hydrogen by the palladium membrane, expansion deformation occurs under the PVDF action, which is converted into voltage signal output. The output voltage of sensors showed a similar change when the sensor was exposed to repeated cycles of H_2_.

**Figure 12:**
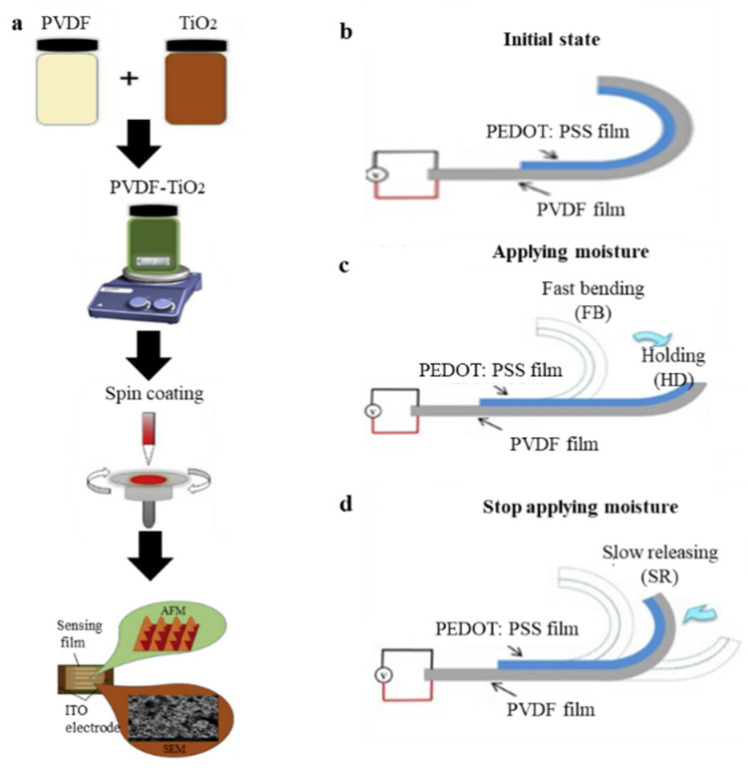
(**a**) Schematic of the spin coated PVDF-TiO_2_ on interdigital ITO electrode [159]; and (**b**–**d**) graphical representation of the PEDOT:PSS/PVDF generator producing a stable DC voltage [160].

The introduction of humidity in sensitive materials can also play a role in humidity sensing. The spin coating technique was used to prepare the PVDF TiO_2_ nanocomposite films on interdigital ITO electrode [159], and the surface morphology was modified by acetone etching. The volume deformation of PVDF is caused by the hygroscopicity of nano-TiO_2_, resulting in a piezoelectric effect (Figure 12a) [159]. Wang et al. [160] proposed a simple and low-cost method to prepare a moisture-responsive composite membrane composed of PEDOT:PSS and PVDF by spin coating and thermal evaporation. The bending angles of the composite film in both directions can reach −191° and 225°, respectively, showing good bidirectional bending performance. They also demonstrated that the bending of the PVDF/ PEDOT:PSS strip at different RHs is recyclable and repeatable. As shown in Figure 12b–d, the PVDF film is glued together with the PEDOT:PSS film. PEDOT:PSS easily absorbs water [160,161,162,163]. In different humidity environments, PEDOT has different bending degrees due to different water absorption rates. PVDF films produce voltage signals under the corresponding bending degrees, representing the specific humidity.

### 4.4. Biosensor and Bionic Actuator Based on Piezoelectric Polymer

**Figure 13:**
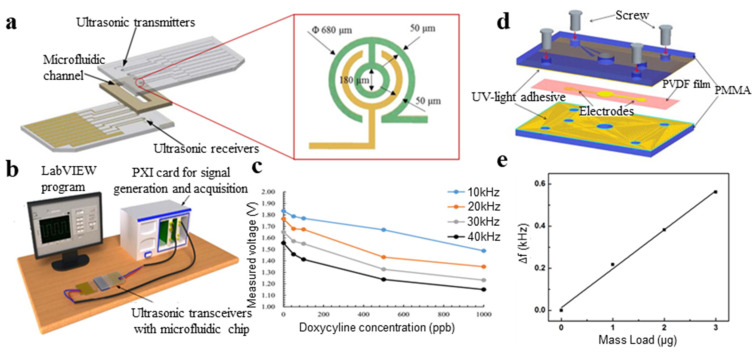
(**a**) Schematic of the chip [164]; (**b**) the experimental setup for characterizing the ultrasonic transceivers [164]; (**c**) the plastic microfluidic chip with ultrasonic transceivers [164]; (**d**) schematic of the biosensor using PVDF film as piezoelectric layer [166]; and (**e**) relationship between mass load and frequency shift (Δf) [166].

Piezoelectric polymers are used as biosensors mainly through biosensor surface modification. The binding of biological target molecules on the sensing surface changes the mass of the sensing end of the sensor, thus affecting the resonant output of the piezoelectric polymer. PVDF-TrFE films were fabricated by electrodeposition, and piezoelectric polymer ultrasonic transceivers were developed, which proved that they can be used for the ultra-sensitive detection of antibiotics. The diameter of the entire ultrasonic transceiver was 680 μm. The ultrasonic transducers consist of a receiver and a transmitter, which is patterned on a gold electrode and integrated with a microfluidic channel. The biosensor was successfully applied to detect the animals’ doxycycline with a detection limit of 50 ppb (Figure 13a–c) [164]. In the field of microfluidic channels, the developed on-chip ultrasonic transducer performed excellently, with the potential to be used for ultra-sensitive detection of DNA, proteins and food antibiotics. Lin et al. [165] fabricated microporous PVDF membranes with different surface morphologies from coagulation baths of different strengths by immersion-precipitation. Using the dual-step procedure, ss-DNA was covalently immobilized on the membranes. The antibody in serum can be effectively adsorbed by the immobilized DNA, performing antibody detection. Similarly, nucleic acid sensors based on PVDF [166] have been developed. In the experiment, they used the hybridization between the capture probe and target analyte (Figure 13d) [166] and found that the mass load on the membrane was proportional to the quantity of target nucleic acids (Figure 13e) [166]. 

**Figure 14:**
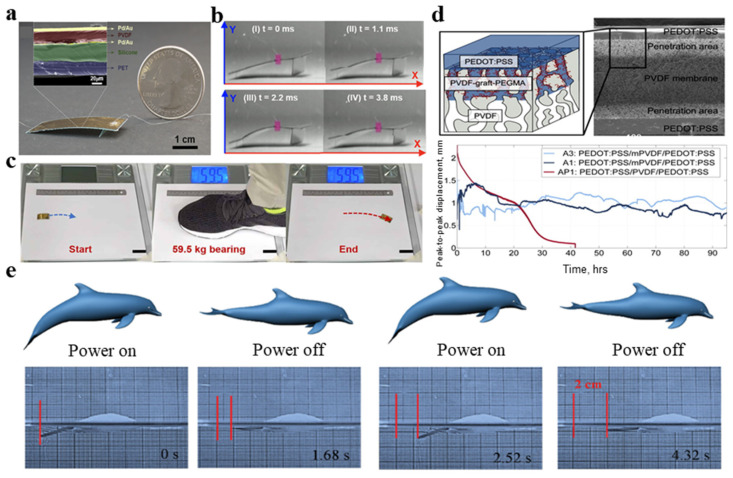
(**a**) Optical photo of the fabricated robot and the SEM image of the cross-sectional view of the prototype robot with different layers of materials [167]; (**b**) images of prototype robot the movements in a driving cycled [167]; (**c**) after bearing the weight of an adult’s footsteps (about one million times heavier than the robot), the system can continue to move [167]; (**d**) the cross-sectional view of the hydrophilic PVDF-graft-PEGMA [168]; and (**e**) a fish-like robot to imitate fish swimming [169].

In recent years, much attention has been paid to the research of artificial bionics devices. In 2019, Wu et al. [167] reported a flexible robot based on a piezoelectric single chip bending structure (Figure 14a,b) [167]. Its relative speed is 20 times its body length per second, which is the fastest measurement speed among the reported artificial robots on an insect scale. The soft robot uses the principle of several kinds of animal movements, such as carrying load, the firmness of a cockroach and climbing slopes. Even after bearing the weight of an adult’s footsteps (approximately 1 million times heavier than the robot), the system can continue to move (Figure 14c) [167].

Piezoelectric polymers also have good performance in the manufacturing of artificial muscles. Simaite et al. [168] prepared a hybrid membrane with hydrophilic PVDF grafted poly(ethylene glycol) monomethyl ether methacrylate (PEGMA) outer surface and hydrophobic main body, which is a model human muscle composition, as shown in Figure 14d. The results are expected to be used in artificial muscles. Xiao et al. [169] fabricated a fish-like robot to imitate fish swimming. The fish-like robot is composed of a body and a tail. The body is made of expandable polystyrene and the tail is made of graphene-PVDF as an actuator. The graphene-PVDF film is connected with a flexible Au wire. When the power is turned on, the fish’s tail is bent, and, when the power is turned off, the tail returns to its original position. As the power is continuously turned on and off, the fish-shaped robot can move forward at a speed of 5.02 mm/s (Figure 14e). Kim et al. [170] fabricated a new IPMC actuator on the basis of blend membrane made by PVDF/polyvinyl pyrrolidone/polystyrene sulfuric acid. The robot can support 2-, 4-, or 6-IPMC-leg models and this miniature walking robot has a size of 18 mm × 11 mm × 12 mm, a weight of 1.3 g and a maximum speed of 0.58 mm/s. 

## 5. Conclusion and Perspectives

Although inorganic piezoelectric materials have the advantages of a large piezoelectric coefficient and high strength, they are fragile and unsuitable for applications with higher flexibility requirements. As a typical representative of flexible piezoelectric polymers, PVDF has irreplaceable advantages in the development of piezoelectric materials. It has great flexibility and its piezoelectric properties can also be improved by doping optimization or other processes. Various doping methods for PVDF polymer materials have been introduced, and the effects of different piezoelectric materials, nanomaterials and biomaterials are compared. Some preparation methods for improving the polarization properties of piezoelectric polymers are also introduced in detail. Based on flexible piezoelectric polymer materials, we can obtain flexible electromechanical devices for different purposes, such as energy harvesting devices, chemical sensor for humidity, pressure, gas and biomolecules. 

We can prepare actuators of different sizes and functions based on flexible piezoelectric polymer materials. However, the piezoelectric coefficient of flexible piezoelectric polymer materials in general is still less than that of the inorganic piezoelectric material. In the follow-up work, the optimization method of the piezoelectric polymer should continue to be studied and applied to wearable devices. (1) Piezoelectric materials based on biological crystals are one of the materials with great potential. The modification of the unique functional groups of biological materials can be used to improve the vertical piezoelectric coefficient of PVDF. (2) Aiming at shape retention, the method of preparing flexible electromechanical devices with high mechanical voltage conversion efficiency should be further explored. (3) With the help of piezocatalysis of PVDF, it can be used for the pretreatment of chemicals in the development of lab-on-a-chip microsystem. (4) In addition, the use of piezoelectric polymer materials for wearable flexible devices requires a piezoelectric polymer exhibiting better skin affinity and more compatibility with other flexible materials to be suitable for the applications of physiological sensors and implantable devices. Biomaterials and flexible conductive polymers can be introduced to improve its biocompatibility, antifouling ability and the capability of integration with hydrophilic surface. In summary, giving full play to the flexible characteristics of piezoelectric polymers and developing wearable piezoelectric sensors and executable devices will have very broad application prospects.

## Figures and Tables

**Table 1:** The advantages of various polarization methods.

Polarization Methods	Advantages
Electrode poling	High d33 coefficient and reproducibility
Corona poling	High d33 coefficient and No requirement for one end structure of the material
Additive manufacturing	Increased the design scope of the three-dimensional structure of the material and low temperature
Mechanical drawing	High d33 coefficient and reproducibility
Electron beam poling	Increased the design scope of the three-dimensional structure of the material

**Table 2:** Fabrication methods, polarization, description and comparison of the performance of PVDF.

Material	Fabrication Method	Poling Conditions	Piezoelectric Coefficient	β-Phase Content (%)	Peak to Peak Voltage	Sensitivity	Maximum Electro Deformation	Application
BTO/P(VDF-TrFE) [90]	Electro spinning	No poling	d33 50 pC/N	81%	-	5 PC/kPa	-	sensing strain
PVDF [134]	3D print	No poling	d31 18 pC/N	64%	Vp-p = 6.3 V	~1.8 V/N	-	
PVDF/AG [99]	Electro spinning	No poling	-	44.50%	Vp-p = 4.6 V	2.5 V (slow and light breathing)	-	
PVDF [133]	3D print	No poling	d33 −8.7 pC/N	61.52%	Vp-p = 0.4 V	-	-	
PVDF/PZT [54]	Solvent Casting	No poling	d33 60–84 pC/N	75%	-	-	-	
PVDF-TrFE [122]	Solvent Casting	Electrode poling (75 MV/m)	d33 20 pC/N	-	-	0.57 pC/g	-	
PVDF-TrFE [121]	Spin coating	No poling	d33 38–74 pC/N	higher with thin film	-	2.35 mV /mmHg	-	
PVDF-TrFE [120]	Spin coating	DC poling (30~60 V)	d33 72.7 pC/N	-	526 mV /a nanopillar	-	-	
PVDF with PDMS/Ag NWs [130]	Electro spinning	No poling	-	57%	Vp-p = 2.2 V	0.02 V/kPa	-	
PVDF /BaTiO_3_ [136]	3D print	Thermal poling (90 ℃ for 2 h)	d31 2.1 × 10E–3 pC/N	61.20%	-	-	-	energy harvesting
PVDF [135]	3D print	Corona poling (280 MV/m)	d31 4.9 × 10E–2 pC/N	56.83%	-	-	-	
PVDF /BT /Graphene [132]	Electro spinning	No poling	-	91.50%	Vp-p = 11 V	-	-	
PVDF/BNT-ST [9]	Electro spinning	No Poling	-	-	Vp-p = 1.31 V	-	-	
PVDF [137]	3D print	No Poling	d33 −33 pC/N	-	Vp-p = 0.3 V	13.3 mV/N	2.02 μm at 860 V	electro deformation
PVDF/CNT [138]	Electro spinning	No Poling	-	90%	Vp-p = 5 V	0.9 V/N	18 μm at 300 V	
PVDF [139]	Spin coating	DC poling (70 MV/m)	-	-	-	-	170 μm (3 mm, at 300 V)	

**Table 3:** Different fabrication methods, fabricated material and sensitivity of various sensors.

Application	Material	Fabrication Methods	Sensitivity
Energy Harvesting Device	PVDF/BT [142]	Electrospinning	power density of 87 μW cm^−3^
	PVDF-TrFE/BNT-ST [143]	Electrospinning.	0.004 V/kPa
	PVDF/SM-KNN [141]	Electrospinning	power density of 115.5 mW/cm^2^
Pressure sensor	PVDF/PDMS-Ag NWS [130]	Electrospinning	0.03 V/kPa
	PVDF/ZNO [87]	Spin-coating and poling	Pressure changes with the lowest value of 10 Pa
	PVDF/BTO [116]	Spin-coating and poling	0.056 V/N
Gas sensor	PVDF/Pd [158]	Polymer film is coated with thin films of Pd on both sides	0.3 V/1% hydrogen concentration
Humidity sensor	PVDF/TiO_2_ [159]	PVDF TiO_2_ nanocomposite films by spin coating	0.02 PF/1% RH
	PVDF/PEDOT: PSS [160]	composite film by spin- coating and thermal evaporation	5°/1% RH
Biosensor	PVDF-TRFE [164]	electrodeposited PVDF-TrFE on the electrode, and modified with chitin on the biosensor surface	detection limit of 50 ppb of Antibiotic
	PVDF [166]	capture probe immobilized on the PVDF film of the biosensor	0.2 kHz/μg

## References

[1] Jaffe H. (1958). Piezoelectric Ceramics. J. Am. Ceram. Soc..

[2] Rao K.S., Sateesh J., Guha K., Baishnab K.L., Ashok P., Sravani K.G. (2018). Design and analysis of MEMS based piezoelectric micro pump integrated with micro needle. Microsyst. Technol..

[3] Liu J., Zuo H., Xia W., Luo Y., Yao D., Chen Y., Wang K., Li Q. (2020). Wind energy harvesting using piezoelectric macro fiber composites based on flutter mode. Microelectron. Eng..

[4] Kaneko R., Froemel J., Tanaka S. (2018). Development of PVDF-TrFE/SiO_2_ composite film bulk acoustic resonator. Sens. Actuators A Phys..

[5] Wang Y., Zhu X., Zhang T., Bano S., Pan H., Qi L., Zhang Z., Yuan Y. (2018). A renewable low-frequency acoustic energy harvesting noise barrier for high-speed railways using a Helmholtz resonator and a PVDF film. Appl. Energy.

[6] Kaneko R., Froemel J., Tanaka S. PVDF—TrFE/SiO_2_ Composite Film Bulk Acoustic Resonator for Frequency-Modulated Sensor Application. Proceedings of the 2018 IEEE International Ultrasonics Symposium (IUS).

[7] Lee H.Y., Choi B. (2013). A multilayer PVDF composite cantilever in the Helmholtz resonator for energy harvesting from sound pressure. Smart Mater. Struct..

[8] Li B., Laviage A.J., You J.H., Kim Y.-J. (2013). Harvesting low-frequency acoustic energy using multiple PVDF beam arrays in quarter-wavelength acoustic resonator. Appl. Acoust..

[9] Ji S.H., Cho J.H., Jeong Y.-H., Paik J.-H., Yun J.D., Yun J.S. (2016). Flexible lead-free piezoelectric nanofiber composites based on BNT-ST and PVDF for frequency sensor applications. Sens. Actuators A Phys..

[10] Koç M., Paralı L., Şan O. (2020). Fabrication and vibrational energy harvesting characterization of flexible piezoelectric nanogenerator (PEN) based on PVDF/PZT. Polym. Test..

[11] Chamankar N., Khajavi R., Yousefi A.A., Rashidi A., Golestani-Fard F. (2020). A flexible piezoelectric pressure sensor based on PVDF nanocomposite fibers doped with PZT particles for energy harvesting applications. Ceram. Int..

[12] Yadav P., Raju T.D., Badhulika S. (2020). Self-Poled hBN-PVDF Nanofiber Mat-Based Low-Cost, Ultrahigh-Performance Piezoelectric Nanogenerator for Biomechanical Energy Harvesting. ACS Appl. Electron. Mater..

[13] Polat K. (2020). Energy harvesting from a thin polymeric film based on PVDF-HFP and PMMA blend. Appl. Phys. A.

[14] Shehata N., Hassanin A.H., Elnabawy E., Nair R., Bhat S.A., Kandas I. (2020). Acoustic Energy Harvesting and Sensing via Electrospun PVDF Nanofiber Membrane. Sensors.

[15] Zhang Q., Sanchez-Fuentes D., Desgarceaux R., Escofet-Majoral P., Oró-Soler J., Gàzquez J., Larrieu G., Charlot B., Gómez A., Gich M. (2019). Micro/Nanostructure Engineering of Epitaxial Piezoelectric α-Quartz Thin Films on Silicon. ACS Appl. Mater. Interfaces.

[16] Zhang H., Yao Y., Shi Y. (2018). Performance Enhancement of Interdigital Electrode-Piezoelectric Quartz Crystal (IDE-PQC) Salt Concentration Sensor by Increasing the Electrode Area of Piezoelectric Quartz Crystal (PQC). Sensors.

[17] Shibata K., Wang R., Tou T., Koruza J. (2018). Applications of lead-free piezoelectric materials. MRS Bull..

[18] Panda P.K. (2009). Review: Environmental friendly lead-free piezoelectric materials. J. Mater. Sci..

[19] Kim H.S., Kim J.-H., Kim J. (2011). A review of piezoelectric energy harvesting based on vibration. Int. J. Precis. Eng. Manuf..

[20] Polsongkram D., Chamninok P., Pukird S., Chow L., Lupan O., Chai G., Khallaf H., Park S., Schulte A. (2008). Effect of synthesis conditions on the growth of ZnO nanorods via hydrothermal method. Phys. B Condens. Matter.

[21] Li H., Tian C., Deng Z.D. (2014). Energy harvesting from low frequency applications using piezoelectric materials. Appl. Phys. Rev..

[22] Khadtare S., Ko E.J., Kim Y.H., Lee H.S., Moon D.K. (2019). A flexible piezoelectric nanogenerator using conducting polymer and silver nanowire hybrid electrodes for its application in real-time muscular monitoring system. Sens. Actuators A Phys..

[23] Pan Q., Xiong Y.-A., Sha T.-T., You Y.-M. (2020). Recent progress in the piezoelectricity of molecular ferroelectrics. Mater. Chem. Front..

[24] Liang Z., Yan C.-F., Rtimi S., Bandara J. (2019). Piezoelectric materials for catalytic/photocatalytic removal of pollutants: Recent advances and outlook. Appl. Catal. B Environ..

[25] Shi J., Zeng W., Dai Z., Wang L., Wang Q., Lin S., Xiong Y., Yang S., Shang S., Chen W. (2020). Piezocatalytic Foam for Highly Efficient Degradation of Aqueous Organics. Small Sci..

[26] Xin Y., Sun H., Tian H., Guo C., Li X., Wang S., Wang C. (2016). The use of polyvinylidene fluoride (PVDF) films as sensors for vibration measurement: A brief review. Ferroelectrics.

[27] Qin C., Gu Y., Sun X., Wang X., Zhang Y. (2015). Structural dependence of piezoelectric size effects and macroscopic polarization in ZnO nanowires: A first-principles study. Nano Res..

[28] Zheng D., Roumanille P., Hermet P., Cambon M., Haines J., Cambon O. (2020). Enhancement of the piezoelectric effect in Fe-substituted GaAsO_4_: A combined XRD, Raman spectroscopy and first principles study. Solid State Sci..

[29] Guo L., Lu Q. (2017). Potentials of piezoelectric and thermoelectric technologies for harvesting energy from pavements. Renew. Sustain. Energy Rev..

[30] Invernizzi F., Dulio S., Patrini M., Guizzetti G., Mustarelli P. (2016). Energy harvesting from human motion: Materials and techniques. Chem. Soc. Rev..

[31] Zhang Q.M., Bharti V., Kavarnos G., Schwartz M., Schwartz M. (2002). Poly(Vinylidene Fluoride) (PVDF) and its Copolymers. The Encyclopedia of Smart Materials.

[32] Martins P., Lopes A.C., Lanceros-Mendez S. (2014). Electroactive phases of poly(vinylidene fluoride): Determination, processing and applications. Prog. Polym. Sci..

[33] Améduri B. (2009). From Vinylidene Fluoride (VDF) to the Applications of VDF-Containing Polymers and Copolymers: Recent Developments and Future Trends. Chem. Rev..

[34] Shepelin N.A., Glushenkov A.M., Lussini V.C., Fox P.J., Dicinoski G.W., Shapter J.G., Ellis A.V. (2019). New developments in composites, copolymer technologies and processing techniques for flexible fluoropolymer piezoelectric generators for efficient energy harvesting. Energy Environ. Sci..

[35] Fang J., Wang X., Lin T. (2011). Electrical power generator from randomly oriented electrospun poly(vinylidene fluoride) nanofibre membranes. J. Mater. Chem..

[36] Huang F., Wei Q., Cai Y., Wu N. (2008). Surface Structures and Contact Angles of Electrospun Poly(vinylidene fluoride) Nanofiber Membranes. Int. J. Polym. Anal. Charact..

[37] Pan C.-T., Yen C.-K., Wang S.-Y., Lai Y.-C., Lin L., Huang J.C., Kuo S.-W. (2015). Near-field electrospinning enhances the energy harvesting of hollow PVDF piezoelectric fibers. RSC Adv..

[38] Liu Z.H., Pan C.T., Lin L.W., Huang J.C., Ou Z.Y. (2014). Direct-write PVDF nonwoven fiber fabric energy harvesters via the hollow cylindrical near-field electrospinning process. Smart Mater. Struct..

[39] Zhu G., Zeng Z., Zhang L., Yan X. (2008). Piezoelectricity in β-phase PVDF crystals: A molecular simulation study. Comput. Mater. Sci..

[40] Hoeher R., Raidt T., Novak N., Katzenberg F., Tiller J.C. (2015). Shape-Memory PVDF Exhibiting Switchable Piezoelectricity. Macromol. Rapid Commun..

[41] Chang C., Tran V.H., Wang J., Fuh Y.-K., Lin L. (2010). Direct-Write Piezoelectric Polymeric Nanogenerator with High Energy Conversion Efficiency. Nano Lett..

[42] Persano L., Dagdeviren C., Su Y., Zhang Y., Girardo S., Pisignano D., Huang Y., Rogers J.A. (2013). High performance piezoelectric devices based on aligned arrays of nanofibers of poly(vinylidenefluoride-co-trifluoroethylene). Nat. Commun..

[43] Pi Z., Zhang J., Wen C., Zhang Z.-B., Wu D. (2014). Flexible piezoelectric nanogenerator made of poly(vinylidenefluoride-co-trifluoroethylene) (PVDF-TrFE) thin film. Nano Energy.

[44] Zhang L., Yu X., You S., Liu H., Zhang C., Cai B., Xiao L., Liu W., Guo S.-S., Zhao X. (2015). Highly sensitive microfluidic flow sensor based on aligned piezoelectric poly(vinylidene fluoride-trifluoroethylene) nanofibers. Appl. Phys. Lett..

[45] Choi K., Lee S.C., Liang Y., Kim K.J., Lee H.S. (2013). Transition from Nanorod to Nanotube of Poly(vinylidene trifluoroethylene) Ferroelectric Nanofiber. Macromolecules.

[46] Gui J., Zhu Y., Zhang L., Shu X., Liu W., Guo S.-S., Zhao X. (2018). Enhanced output-performance of piezoelectric poly(vinylidene fluoride trifluoroethylene) fibers-based nanogenerator with interdigital electrodes and well-ordered cylindrical cavities. Appl. Phys. Lett..

[47] Oh S.R., Yao K., Chow C.L., Tay F.E.H. (2010). Residual stress in piezoelectric poly(vinylidene-fluoride-co-trifluoroethylene) thin films deposited on silicon substrates. Thin Solid Films.

[48] Shao H., Fang J., Wang H., Lin T. (2015). Effect of electrospinning parameters and polymer concentrations on mechanical-to-electrical energy conversion of randomly-oriented electrospun poly(vinylidene fluoride) nanofiber mats. RSC Adv..

[49] Lang C., Fang J., Shao H., Wang H., Yan G., Ding X., Lin T. (2017). High-output acoustoelectric power generators from poly(vinylidenefluoride-co-trifluoroethylene) electrospun nano-nonwovens. Nano Energy.

[50] Soin N., Shah T.H., Anand S.C., Geng J., Pornwannachai W., Mandal P., Reid D.G., Sharma S., Hadimani R., Bayramol D.V. (2014). Novel “3-D spacer” all fibre piezoelectric textiles for energy harvesting applications. Energy Environ. Sci..

[51] Zirkl M., Sawatdee A., Helbig U., Krause M., Scheipl G., Kraker E., Ersman P.A., Nilsson D., Platt D., Bodö P. (2011). An All-Printed Ferroelectric Active Matrix Sensor Network Based on Only Five Functional Materials Forming a Touchless Control Interface. Adv. Mater..

[52] Yuan Y., Reece T.J., Sharma P., Poddar S., Ducharme S., Gruverman A., Yang Y., Huang J. (2011). Efficiency enhancement in organic solar cells with ferroelectric polymers. Nat. Mater..

[53] Kang S.J., Bae I., Shin Y.J., Park Y.J., Huh J., Park S.-M., Kim H.-C., Park C. (2011). Nonvolatile Polymer Memory with Nanoconfinement of Ferroelectric Crystals. Nano Lett..

[54] Tiwari V., Srivastava G. (2015). Structural, dielectric and piezoelectric properties of 0–3 PZT/PVDF composites. Ceram. Int..

[55] Luo C., Yang C.-W., Cao G.Z., Shen I.Y., Tai W.C. (2016). Effects of added mass on lead-zirconate-titanate (pzt) thin-film microactuators in aqueous environments. J. Vib. Acoust..

[56] Pal A., Sasmal A., Manoj B., PrasadaRao D.S.D., Haldar A.K., Sen S. (2020). Enhancement in energy storage and piezoelectric performance of three phase (PZT/MWCNT/PVDF) composite. Mater. Chem. Phys..

[57] Sabry R.S., Hussein A.D. (2019). PVDF: ZnO/BaTiO_3_ as high out-put piezoelectric nanogenerator. Polym. Test..

[58] Sasmal A., Sen S., Devi P.S. (2020). Frequency dependent energy storage and dielectric performance of Ba–Zr Co-doped BiFeO_3_ loaded PVDF based mechanical energy harvesters: Effect of corona poling. Soft Matter.

[59] Li R., Zhao Z., Chen Z., Pei J. (2017). Novel BaTiO_3_/PVDF composites with enhanced electrical properties modified by calcined BaTiO_3_ ceramic powders. Mater. Express.

[60] Kakimoto K., Fukata K., Ogawa H. (2013). Fabrication of fibrous BaTiO_3_-reinforced PVDF composite sheet for transducer application. Sens. Actuators A Phys..

[61] Alluri N.R., Saravanakumar B., Kim S.-J. (2015). Flexible, Hybrid Piezoelectric Film (BaTi(1–x)ZrxO_3_)/PVDF Nanogenerator as a Self-Powered Fluid Velocity Sensor. ACS Appl. Mater. Interfaces.

[62] Krauss W., Schütz D., Mautner F.A., Feteira A., Reichmann K. (2010). Piezoelectric properties and phase transition temperatures of the solid solution of (1−x)(Bi0.5Na0.5)TiO_3_–xSrTiO_3_. J. Eur. Ceram. Soc..

[63] Yu H., Ye Z.-G. (2008). Dielectric, ferroelectric, and piezoelectric properties of the lead-free (1−x)(Na0.5Bi0.5)TiO_3_-xBiAlO_3_ solid solution. Appl. Phys. Lett..

[64] Jiang Y., Qin B., Zhao Y., Jiang Y., Shi W., Li Q., Xiao D., Zhu J. (2008). Phase Transition, Piezoelectric Properties, and Thermal Stability of (1−x−y)BiScO_3_-yBiGaO_3_-xPbTiO_3_ Ceramics. J. Am. Ceram. Soc..

[65] Choi S.M., Stringer C.J., Shrout T.R., Randall C.A. (2005). Structure and property investigation of a Bi-based perovskite solid solution: (1−x)Bi(Ni1/2Ti1/2)O_3_–xPbTiO_3_. J. Appl. Phys..

[66] Lin D., Kwok K.W. (2009). Structure, ferroelectric and piezoelectric properties of (Bi0.98−x La0.02Na1−x )0.5Ba x TiO_3_ lead-free ceramics. Appl. Phys. A.

[67] Fu H., Cohen R.E. (2000). Polarization rotation mechanism for ultrahigh electromechanical response in single-crystal piezoelectrics. Nature.

[68] Karanth D., Fu H. (2005). Large electromechanical response in ZnO and its microscopic origin. Phys. Rev. B.

[69] Hernandez B.A., Chang K.-S., Fisher A.E.R., Dorhout P.K. (2002). Sol−Gel Template Synthesis and Characterization of BaTiO_3_ and PbTiO_3_ Nanotubes. Chem. Mater..

[70] Wang X., Li Y. (2002). Synthesis and Characterization of Lanthanide Hydroxide Single-Crystal Nanowires. Angew. Chem. Int. Ed..

[71] Scott J.F. (2007). Applications of Modern Ferroelectrics. Science.

[72] Webb J.F. (2003). A General Approach to Perturbation Theoretic Analysis in Nonlinear Optics and its Application to Ferroelectrics and Antiferroelectrics. Int. J. Mod. Phys. B.

[73] Kalyani A.K., Senyshyn A., Ranjan R. (2013). Polymorphic phase boundaries and enhanced piezoelectric response in extended composition range in the lead free ferroelectric BaTi1−xZrxO_3_. J. Appl. Phys..

[74] Chen M., Xu Z., Chu R., Liu Y., Shao L., Li W., Gong S., Li G. (2013). Polymorphic phase transition and enhanced piezoelectric properties in (Ba0.9Ca0.1)(Ti1−xSnx)O_3_ lead-free ceramics. Mater. Lett..

[75] Yu Z., Ang C., Guo R., Bhalla A. (2002). Piezoelectric and strain properties of Ba(Ti1−xZrx)O3 ceramics. J. Appl. Phys..

[76] Rachakom A., Jiansirisomboon S., Watcharapasorn A. (2014). Effect of poling on piezoelectric and ferroelectric properties of Bi0.5Na0.5Ti1-xZrxO_3_ ceramics. J. Electroceram..

[77] Kim J., Loh K.J., Lynch J.P., Tomizuka M. (2008). Piezoelectric polymeric thin films tuned by carbon nanotube fillers. Sensors and Smart Structures Technologies for Civil, Mechanical, and Aerospace Systems 2008, Pts 1 and 2.

[78] El Achaby M., Arrakhiz F., Vaudreuil S., Essassi E.M., Qaiss A. (2012). Piezoelectric β-polymorph formation and properties enhancement in graphene oxide—PVDF nanocomposite films. Appl. Surf. Sci..

[79] Batth A., Mueller A., Rakesh L., Mellinger A. Electrical properties of poly(vinylidene fluoride-hexafluoropropylene) (PVDF-HFP) blended with carbon nanotubes. Proceedings of the Electrical Insulation & Dielectric Phenomena.

[80] Rahman A., Lee B.-C., Phan D.-T., Chung G.-S. (2013). Fabrication and characterization of highly efficient flexible energy harvesters using PVDF–graphene nanocomposites. Smart Mater. Struct..

[81] Huang L., Lu C., Wang F., Wang L. (2014). Preparation of PVDF/graphene ferroelectric composite films by in situ reduction with hydrobromic acids and their properties. RSC Adv..

[82] Shetty S., Mahendran A., Anandhan S. (2020). Development of a new flexible nanogenerator from electrospun nanofabric based on PVDF/talc nanosheet composites. Soft Matter.

[83] Sun D., Chang C., Li S., Lin L. (2006). Near-Field Electrospinning. Nano Lett..

[84] Di Camillo D., Fasano V., Ruggieri F., Santucci S., Lozzi L., Camposeo A., Pisignano D. (2013). Near-field electrospinning of light-emitting conjugated polymer nanofibers. Nanoscale.

[85] Yun J.S., Kil Park C., Jeong Y.-H., Cho J.H., Paik J.-H., Yoon S.H., Hwang K.-R. (2016). The Fabrication and Characterization of Piezoelectric PZT/PVDF Electrospun Nanofiber Composites. Nanomater. Nanotechnol..

[86] Yun J.S., Kil Park C., Cho J.H., Paik J.-H., Jeong Y.H., Nam J.-H., Hwang K.-R. (2014). The effect of PVP contents on the fiber morphology and piezoelectric characteristics of PZT nanofibers prepared by electrospinning. Mater. Lett..

[87] Lee J.S., Shin K.-Y., Cheong O.J., Kim J.H., Jang J. (2015). Highly Sensitive and Multifunctional Tactile Sensor Using Free-standing ZnO/PVDF Thin Film with Graphene Electrodes for Pressure and Temperature Monitoring. Sci. Rep..

[88] Shin K.-Y., Lee J.S., Jang J. (2016). Highly sensitive, wearable and wireless pressure sensor using free-standing ZnO nanoneedle/PVDF hybrid thin film for heart rate monitoring. Nano Energy.

[89] Xin Y., Qi X., Tian H., Guo C., Li X., Lin J., Wang C. (2016). Full-fiber piezoelectric sensor by straight PVDF/nanoclay nanofibers. Mater. Lett..

[90] Hu X., Yan X., Gong L., Wang F., Xu Y., Feng L., Zhang D., Jiang Y. (2019). Improved Piezoelectric Sensing Performance of P(VDF–TrFE) Nanofibers by Utilizing BTO Nanoparticles and Penetrated Electrodes. ACS Appl. Mater. Interfaces.

[91] Wu C.-M., Chou M.H. (2016). Polymorphism, piezoelectricity and sound absorption of electrospun PVDF membranes with and without carbon nanotubes. Compos. Sci. Technol..

[92] Hosseini S.M., Yousefi A.A. (2017). Piezoelectric sensor based on electrospun PVDF-MWCNT-Cloisite 30B hybrid nanocomposites. Org. Electron..

[93] Lins L.C., Wianny F., Livi S., Dehay C., Duchet-Rumeau J., Gérard J.-F. (2017). Effect of polyvinylidene fluoride electrospun fiber orientation on neural stem cell differentiation. J. Biomed. Mater. Res. Part B Appl. Biomater..

[94] Zheng J., He A., Li J., Han C.C. (2007). Polymorphism Control of Poly(vinylidene fluoride) through Electrospinning. Macromol. Rapid Commun..

[95] Pu J., Yan X., Jiang Y., Chang C., Lin L. (2010). Piezoelectric actuation of direct-write electrospun fibers. Sens. Actuators A Phys..

[96] Khalifa M., Deeksha B., Mahendran A., Anandhan S. (2018). Synergism of Electrospinning and Nano-alumina Trihydrate on the Polymorphism, Crystallinity and Piezoelectric Performance of PVDF Nanofibers. JOM.

[97] Lei T., Yu L., Wang L., Yang F., Sun D. (2015). Predicting Polymorphism of Electrospun Polyvinylidene Fluoride Membranes by Their Morphologies. J. Macromol. Sci. Part B.

[98] Sinha T.K., Ghosh S.K., Maiti R., Jana S., Adhikari B., Mandal D., Ray S.K. (2016). Graphene-Silver-Induced Self-Polarized PVDF-Based Flexible Plasmonic Nanogenerator Toward the Realization for New Class of Self Powered Optical Sensor. ACS Appl. Mater. Interfaces.

[99] Li Y., Zheng Y., Liu Z., Li J., Zhai H., Chen Z., Li Y. (2020). Design of an Ultrasensitive Flexible Bend Sensor Using a Silver-Doped Oriented Poly(vinylidene fluoride) Nanofiber Web for Respiratory Monitoring. ACS Appl. Mater. Interfaces.

[100] Das A., Pisana S., Chakraborty B., Piscanec S., Saha S.K., Waghmare U.V., Novoselov K.S., Krishnamurthy H.R., Geim A.K., Ferrari A.C. (2008). Monitoring dopants by Raman scattering in an electrochemically top-gated graphene transistor. Nat. Nanotechnol..

[101] Shi Y., Dong X., Chen P., Wang J., Li L.-J. (2009). Effective doping of single-layer graphene from underlying SiO_2_ substrates. Phys. Rev. B.

[102] Losurdo M., Bergmair I., Dastmalchi B., Kim T.-H., Giangregroio M.M., Jiao W., Bianco G.V., Brown A.S., Hingerl K., Bruno G. (2014). Graphene as an Electron Shuttle for Silver Deoxidation: Removing a Key Barrier to Plasmonics and Metamaterials for SERS in the Visible. Adv. Funct. Mater..

[103] Maiti R., Sinha T.K., Mukherjee S., Adhikari B., Katiyar A.K. (2016). Enhanced and Selective Photodetection Using Graphene-Stabilized Hybrid Plasmonic Silver Nanoparticles. Plasmonics.

[104] Kravets V.G., Jalil R., Kim Y.-J., Ansell D., Aznakayeva D.E., Thackray B., Britnell L., Belle B.D., Withers F., Radko I.P. (2015). Graphene-protected copper and silver plasmonics. Sci. Rep..

[105] Gan W.C., Majid W.H.A. (2014). Effect of TiO_2_ on enhanced pyroelectric activity of PVDF composite. Smart Mater. Struct..

[106] Al-Saygh A., Ponnamma D., Almaadeed M.A., Vijayan P P., Karim A., Hassan M.K. (2017). Flexible Pressure Sensor Based on PVDF Nanocomposites Containing Reduced Graphene Oxide-Titania Hybrid Nanolayers. Polymers.

[107] Norden A.G., Lapsley M., Lee P.J., Pusey C.D., Scheinman S.J., Tam F.W., Thakker R., Unwin R.J., Wrong O. (2001). Glomerular protein sieving and implications for renal failure in Fanconi syndrome. Kidney Int..

[108] Mishra M., Roy A., Dash S., Mukherjee S., Mondal A.K., Mallik A. Flexible nano-GFO/PVDF piezoelectric-polymer nano-composite films for mechanical energy harvesting. Proceedings of the 7th National Conference on Processing and Characterization of Materials.

[109] Jaleh B., Jabbari A. (2014). Evaluation of reduced graphene oxide/ZnO effect on properties of PVDF nanocomposite films. Appl. Surf. Sci..

[110] Dodds J.S., Meyers F.N., Loh K.J. (2011). Piezoelectric Characterization of PVDF-TrFE Thin Films Enhanced With ZnO Nanoparticles. IEEE Sens. J..

[111] Bhunia R., Das S., Dalui S., Hussain S., Paul R., Bhar R., Pal A. (2016). Flexible nano-ZnO/polyvinylidene difluoride piezoelectric composite films as energy harvester. Appl. Phys. A.

[112] Jin C., Hao N., Xu Z., Trase I., Nie Y., Dong L., Closson A., Chen Z., Zhang J.X.J. (2020). Flexible piezoelectric nanogenerators using metal-doped ZnO-PVDF films. Sens. Actuators A Phys..

[113] Nuraeva A.S., Zelenovskiy P.S., Slashchev A., Gruzdev D.A., Slepukhin P.A., Olshevskaya V.A., Krasnov V.P., Shur V.Y. (2017). Morphology and piezoelectric characterization of thin films and microcrystals of ortho-carboranyl derivatives of (S)-glutamine and (S)-asparagine. Ferroelectrics.

[114] Stapleton A., Noor M.R., Sweeney J., Casey V., Kholkin A., Silien C., Gandhi A.A., Soulimane T., Tofail S.A. (2017). The direct piezoelectric effect in the globular protein lysozyme. Appl. Phys. Lett..

[115] Nguyen V., Zhu R., Jenkins K., Yang R. (2016). Self-assembly of diphenylalanine peptide with controlled polarization for power generation. Nat. Commun..

[116] Yang Y., Pan H., Xie G., Jiang Y., Chen C., Su Y., Wang Y., Tai H. (2020). Flexible piezoelectric pressure sensor based on polydopamine-modified BaTiO_3_/PVDF composite film for human motion monitoring. Sens. Actuators A Phys..

[117] Tamang A., Ghosh S.K., Garain S., Alam M., Haeberle J., Henkel K., Schmeisser D., Mandal D. (2015). DNA-Assisted β-phase Nucleation and Alignment of Molecular Dipoles in PVDF Film: A Realization of Self-Poled Bioinspired Flexible Polymer Nanogenerator for Portable Electronic Devices. ACS Appl. Mater. Interfaces.

[118] Ramadan K.S., Sameoto D., Evoy S. (2014). A review of piezoelectric polymers as functional materials for electromechanical transducers. Smart Mater. Struct..

[119] Chen S., Li X., Yao K., Tay F.E.H., Kumar A., Zeng K. (2012). Self-polarized ferroelectric PVDF homopolymer ultra-thin films derived from Langmuir–Blodgett deposition. Polymer.

[120] Hong C.-C., Huang S.-Y., Shieh J., Chen S.-H. (2012). Enhanced Piezoelectricity of Nanoimprinted Sub-20 nm Poly(vinylidene fluoride–trifluoroethylene) Copolymer Nanograss. Macromolecules.

[121] Sharma T., Je S.-S., Gill B.S., Zhang J.X. (2012). Patterning piezoelectric thin film PVDF–TrFE based pressure sensor for catheter application. Sens. Actuators A Phys..

[122] Schulze R., Gessner T., Schueller M., Forke R., Billep D., Heinrich M., Sborikas M., Wegener M., IEEE Integration of Piezoelectric Polymer Transducers into Microsystems for Sensing Applications. Proceedings of the 2012 International Symposium on Applications of Ferroelectrics Held Jointly with 11th IEEE Ecapd and IEEE Pfm.

[123] Mallick S., Ahmad Z., Qadir K.W., Rehman A., Shakoor R.A., Touati F., Al-Muhtaseb S. (2020). Effect of BaTiO_3_ on the sensing properties of PVDF composite-based capacitive humidity sensors. Ceram. Int..

[124] Guo R., Zhang H., Cao S., Cui X., Yan Z., Sang S. (2019). A self-powered stretchable sensor fabricated by serpentine PVDF film for multiple dynamic monitoring. Mater. Des..

[125] Ha T., Tran J., Liu S., Jang H., Jeong H., Mitbander R., Huh H., Qiu Y., Duong J., Wang R.L. (2019). A Chest-Laminated Ultrathin and Stretchable E-Tattoo for the Measurement of Electrocardiogram, Seismocardiogram, and Cardiac Time Intervals. Adv. Sci..

[126] Liu S., Ha T., Lu N. (2019). Experimentally and Numerically Validated Analytical Solutions to Nonbuckling Piezoelectric Serpentine Ribbons. J. Appl. Mech..

[127] Sun R., Zhang B., Yang L., Zhang W., Farrow I., Scarpa F., Rossiter J. (2018). Kirigami stretchable strain sensors with enhanced piezoelectricity induced by topological electrodes. Appl. Phys. Lett..

[128] Jain A., Prashanth K., Sharma A.K., Jain A., Rashmi P.N. (2015). Dielectric and piezoelectric properties of PVDF/PZT composites: A review. Polym. Eng. Sci..

[129] Huang Z., Zhang Y.-Z., Kotaki M., Ramakrishna S. (2003). A review on polymer nanofibers by electrospinning and their applications in nanocomposites. Compos. Sci. Technol..

[130] Wang G., Liu T., Sun X.-C., Li P., Xu Y.-S., Hua J.-G., Yu Y.-H., Li S.-X., Dai Y.-Z., Song X.-Y. (2018). Flexible pressure sensor based on PVDF nanofiber. Sens. Actuators A Phys..

[131] Singh R.K., Lye S.W., Miao J. (2020). Measurement of impact characteristics in a string using electrospun PVDF nanofibers strain sensors. Sens. Actuators A Phys..

[132] Shi K., Sun B., Huang X., Jiang P. (2018). Synergistic effect of graphene nanosheet and BaTiO_3_ nanoparticles on performance enhancement of electrospun PVDF nanofiber mat for flexible piezoelectric nanogenerators. Nano Energy.

[133] Chen C., Cai F., Zhu Y., Liao L., Qian J., Yuan F.-G., Zhang N. (2019). 3D printing of electroactive PVDF thin films with high β-phase content. Smart Mater. Struct..

[134] Bodkhe S., Turcot G., Gosselin F.P., Therriault D. (2017). One-Step Solvent Evaporation-Assisted 3D Printing of Piezoelectric PVDF Nanocomposite Structures. ACS Appl. Mater. Interfaces.

[135] Kim H., Torres F., Wu Y., Villagran D., Lin Y., Tseng T.-L.B. (2017). Integrated 3D printing and corona poling process of PVDF piezoelectric films for pressure sensor application. Smart Mater. Struct..

[136] Kim H., Fernando T., Li M., Lin Y., Tseng T.-L.B. (2018). Fabrication and characterization of 3D printed BaTiO_3_/PVDF nanocomposites. J. Compos. Mater..

[137] Burnham-Fay E.D., Le T., Tarbutton J.A., Ellis J.D. (2017). Strain characteristics of additive manufactured polyvinylidene fluoride (PVDF) actuators. Sens. Actuators A Phys..

[138] Sharafkhani S., Kokabi M. (2020). Ultrathin-Shell PVDF/CNT Nanocomposite Aligned Hollow Fibers as a Sensor/Actuator Single Element. Compos. Sci. Technol..

[139] Gangal, Bodas PVdF based micro actuator. Proceedings of the International Symposium on Physics & Technology of Sensors.

[140] Shaikh M.O., Huang Y.-B., Wang C.-C., Chuang C.-H. (2019). Wearable Woven Triboelectric Nanogenerator Utilizing Electrospun PVDF Nanofibers for Mechanical Energy Harvesting. Micromachines.

[141] Bairagi S., Ali S.W. (2020). Flexible lead-free PVDF/SM-KNN electrospun nanocomposite based piezoelectric materials: Significant enhancement of energy harvesting efficiency of the nanogenerator. Energy.

[142] Mokhtari F., Spinks G.M., Fay C., Cheng Z., Raad R., Xi J., Foroughi J. (2020). Wearable Electronic Textiles from Nanostructured Piezoelectric Fibers. Adv. Mater. Technol..

[143] Ji S.H., Cho Y.-S., Yun J.S. (2019). Wearable Core-Shell Piezoelectric Nanofiber Yarns for Body Movement Energy Harvesting. Nanomaterials.

[144] Implantable Micro-Devices for Self-Charging Pacemakers. https://eepower.com/news/implantable-micro-devices-for-self-charging-pacemakers/.

[145] Yee W.A., Kotaki M., Liu Y., Lu X. (2007). Morphology, polymorphism behavior and molecular orientation of electrospun poly(vinylidene fluoride) fibers. Polymer.

[146] Li D., Xia Y. (2004). Electrospinning of Nanofibers: Reinventing the Wheel?. Adv. Mater..

[147] Li B., Xu C., Zheng J., Xu C. (2014). Sensitivity of Pressure Sensors Enhanced by Doping Silver Nanowires. Sensors.

[148] Matthews J.A., Wnek G.E., Simpson D.G., Bowlin G.L. (2002). Electrospinning of Collagen Nanofibers. Biomacromolecules.

[149] Meng L., Arnoult O., Smith M., Wnek G.E. (2012). Electrospinning of in situ crosslinked collagen nanofibers. J. Mater. Chem..

[150] Je S.-S., Sharma T., Lee Y., Gill B., Zhang J.X., IEEE A thin-film piezoelectric pvdf-trfe based implantable pressure sensor using lithographic patterning. Proceedings of the 2011 IEEE 24th International Conference on Micro Electro Mechanical Systems.

[151] Kim H., Torres F., Villagran D., Stewart C., Lin Y., Tseng T.-L.B. (2017). 3D Printing of BaTiO_3_/PVDF Composites with Electric In Situ Poling for Pressure Sensor Applications. Macromol. Mater. Eng..

[152] Huang L., Lu C., Wang F., Dong X. (2016). Piezoelectric property of PVDF/graphene composite films using 1H, 1H, 2H, 2H-Perfluorooctyltriethoxysilane as a modifying agent. J. Alloy. Compd..

[153] Spanu A., Pinna L., Viola F., Seminara L., Valle M., Bonfiglio A., Cosseddu P. (2016). A high-sensitivity tactile sensor based on piezoelectric polymer PVDF coupled to an ultra-low voltage organic transistor. Org. Electron..

[154] Hsu Y.-J., Jia Z., Kymissis I. (2011). A Locally Amplified Strain Sensor Based on a Piezoelectric Polymer and Organic Field-Effect Transistors. IEEE Trans. Electron. Devices.

[155] Griffiths A.G., Patwari P.P., Loghmanee D.A., Balog M.J., Trosman I., Sheldon S.H. (2017). Validation of Polyvinylidene Fluoride Impedance Sensor for Respiratory Event Classification during Polysomnography in Children. J. Clin. Sleep Med..

[156] Gao S., Guo R., Shao M., Xu L. (2020). A Touch Orientation Classification-Based Force–Voltage Responsivity Stabilization Method for Piezoelectric Force Sensing in Interactive Displays. IEEE Sens. J..

[157] Liu Z., Yang X., Sun J., Ma F. (2018). PVDF modified Pd-SnO_2_ hydrogen sensor with stable response under high humidity. Mater. Lett..

[158] Imai Y., Tadaki D., Ma T., Kimura Y., Hirano-Iwata A., Niwano M. (2017). Response characteristics of hydrogen gas sensor with porous piezoelectric poly(vinylidene fluoride) film. Sens. Actuators B Chem..

[159] Mallick S., Ahmad Z., Touati F., Shakoor R. (2019). Improvement of humidity sensing properties of PVDF-TiO_2_ nanocomposite films using acetone etching. Sens. Actuators B Chem..

[160] Wang G., Xia H., Sun X.-C., Lv C., Li S.-X., Han B., Guo Q., Shi Q., Wang Y.-S., Sun H.-B. (2018). Actuator and generator based on moisture-responsive PEDOT: PSS/PVDF composite film. Sens. Actuators B Chem..

[161] Shen H., Ding J., Yuan N., Xu J., Han L., Zhou X., Fang B. (2018). Flexible Actuator and Generator Stimulated by Organic Vapors. J. Inorg. Organomet. Polym. Mater..

[162] Li S., Chen S., Zhuo B., Li Q., Liu W., Guo X. (2017). Flexible Ammonia Sensor Based on PEDOT:PSS/Silver Nanowire Composite Film for Meat Freshness Monitoring. IEEE Electron. Device Lett..

[163] Cho M.S., Seo H.J., Nam J.-D., Lee Y., Son Y.K. (2007). A Solid State Actuator Based on the PEDOT/NBR System: Effect of Anion Size of Imidazolium Ionic Liquid. Mol. Cryst. Liq. Cryst..

[164] Chen K.-W., Chen G.-L., Hong C.-C. (2016). Electrodeposition of Piezoelectric Polymer Ultrasonic Transceivers for On-Chip Antibiotic Biosensors. J. Electrochem. Soc..

[165] Lin D.-J., Lin D.-T., Young T.-H., Chen T.-C., Chang H.-H., Cheng L.-P. (2009). Immobilization of DNA on Microporous PVDF Membranes by Plasma Polymerization. J. Biomater. Sci. Polym. Ed..

[166] Zhao B., Hu J., Ren W., Xu F., Wu X., Shi P., Ye Z.-G. (2015). A new biosensor based on PVDF film for detection of nucleic acids. Ceram. Int..

[167] Wu Y., Yim J.K., Liang J., Shao Z., Qi M., Zhong J., Luo Z., Yan X., Zhang M., Wang X. (2019). Insect-scale fast moving and ultrarobust soft robot. Sci. Robot..

[168] Simaite A., Tondu B., Souères P., Bergaud C. (2015). Hybrid PVDF/PVDF-graft-PEGMA Membranes for Improved Interface Strength and Lifetime of PEDOT:PSS/PVDF/Ionic Liquid Actuators. ACS Appl. Mater. Interfaces.

[169] Xiao P., Yi N., Zhang T., Huang Y., Chang H., Yang Y., Zhou Y., Chen Y. (2016). Construction of a Fish-like Robot Based on High Performance Graphene/PVDF Bimorph Actuation Materials. Adv. Sci..

[170] Nguyen K.T., Ko S.Y., Park J.-O., Park S. (2016). Miniaturized Terrestrial Walking Robot Using PVDF/PVP/PSSA Based Ionic Polymer–Metal Composite Actuator. J. Mech. Robot..

